# Muscle mass, BMI, and mortality among adults in the United States: A population-based cohort study

**DOI:** 10.1371/journal.pone.0194697

**Published:** 2018-04-11

**Authors:** Matthew K. Abramowitz, Charles B. Hall, Afolarin Amodu, Deep Sharma, Lagu Androga, Meredith Hawkins

**Affiliations:** 1 Department of Medicine, Albert Einstein College of Medicine, Bronx, NY, United States of America; 2 Department of Epidemiology & Population Health, Albert Einstein College of Medicine, Bronx, NY, United States of America; 3 The Saul R. Korey Department of Neurology, Albert Einstein College of Medicine, Bronx, NY, United States of America; 4 Department of Medicine, Brigham and Women's Hospital and Massachusetts General Hospital, Boston, MA, United States of America; Dartmouth College Geisel School of Medicine, UNITED STATES

## Abstract

**Background:**

The level of body-mass index (BMI) associated with the lowest risk of death remains unclear. Although differences in muscle mass limit the utility of BMI as a measure of adiposity, no study has directly examined the effect of muscle mass on the BMI-mortality relationship.

**Methods:**

Body composition was measured by dual-energy x-ray absorptiometry in 11,687 participants of the National Health and Nutrition Examination Survey 1999–2004. Low muscle mass was defined using sex-specific thresholds of the appendicular skeletal muscle mass index (ASMI). Proportional hazards models were created to model associations with all-cause mortality.

**Results:**

At any level of BMI ≥22, participants with low muscle mass had higher body fat percentage (%TBF), an increased likelihood of diabetes, and higher adjusted mortality than other participants. Increases in %TBF manifested as 30–40% smaller changes in BMI than were observed in participants with preserved muscle mass. Excluding participants with low muscle mass or adjustment for ASMI attenuated the risk associated with low BMI, magnified the risk associated with high BMI, and shifted downward the level of BMI associated with the lowest risk of death. Higher ASMI was independently associated with lower mortality. Effects were similar in never-smokers and ever-smokers. Additional adjustment for waist circumference eliminated the risk associated with higher BMI. Results were unchanged after excluding unintentional weight loss, chronic illness, early mortality, and participants performing muscle-strengthening exercises or recommended levels of physical activity.

**Conclusions:**

Muscle mass mediates associations of BMI with adiposity and mortality and is inversely associated with the risk of death. After accounting for muscle mass, the BMI associated with the greatest survival shifts downward toward the normal range. These results provide a concrete explanation for the obesity paradox.

## Introduction

Numerous studies over the past two decades have shown a body-mass index (BMI) in the normal range is associated with the lowest risk of death [1–6]. Other large cohort studies in various populations have reached different conclusions, demonstrating a survival benefit for overweight or even obesity, which has been interpreted by many as a causal relationship [7–12]. The possibility of this “obesity paradox” continues to be debated in the literature and is of great public health importance, not least because of the message communicated to the public.

However, reduced survival for people with normal BMI, compared with overweight, might be explained by loss of muscle mass in the former [13]. In support of this hypothesis, exclusion of people with a history of smoking or chronic disease, both of which promote weight loss and muscle wasting [14–16], lowers the BMI at which long-term survival is greatest [1, 2, 4, 5, 7, 17]. Still, nearly all sizable cohort studies continue to use BMI as the defining metric, and none have systematically examined the impact of muscle mass on the BMI-mortality relationship. As the obesity epidemic spreads throughout the developing world, it is crucial to accurately assess its health impact.

We hypothesized that (1) accounting for persons with low muscle mass would identify distinct populations within BMI categories with disparate risks of death, and (2) that accounting for muscle mass would reduce the level of BMI that was associated with the lowest risk of death and magnify the risk associated with high BMI. We tested these hypotheses using nationally representative data including body composition measurements from participants in the National Health and Nutrition Examination Survey (NHANES).

## Methods

### Study population

NHANES employs a stratified, multistage, probability sampling design to conduct a nationally representative survey of the non-institutionalized civilian population in the United States. The NHANES protocol was approved by the National Center for Health Statistics ethics review board in accordance with the Declaration of Helsinki and written informed consent was obtained from all participants. From 1999–2004, 14,213 adults ≥20 years of age completed the interview and examination components, of whom 12,544 had body composition (BMI and dual-energy x-ray absorptiometry (DEXA)) and mortality data available. Because of the small number of participants with BMI <18.5 kg/m^2^ (n = 201) and because DEXA data were imputed in 55.7% of participants with BMI >40 kg/m^2^ (n = 621), we limited our analysis to those with BMI 18.5–40 kg/m^2^. After excluding 35 individuals with missing data on covariates of interest, the final cohort comprised 11,687 participants.

### Data collection

Race/ethnicity, education, household income, smoking status, physical activity, and comorbidities were determined by self-report. Physical activity (MET-min/wk) was calculated using self-reported frequency and duration of activities such as walking, cycling, home or yard work, and moderate or vigorous leisure activity within the past 30 days [18]. Activity level was categorized as 0, <500, 500–2000, or >2000 MET-min/wk. Participants were considered to have performed muscle strengthening activities if they answered yes to performing physical activities specifically designed to strengthen muscles. Diabetes mellitus was defined as a physician diagnosis while not pregnant, the use of insulin or oral hypoglycemic medications, or a glycohemoglobin level ≥6.5%. Hypertension was defined by systolic blood pressure ≥140 mmHg, diastolic blood pressure ≥90 mmHg, history of physician diagnosis, and/or antihypertensive medication use [19]. Cardiovascular disease (CVD) was defined by physician diagnosis of congestive heart failure, coronary heart disease, angina, myocardial infarction, or stroke. Participants were asked to report their weight at the time of examination and 1-year prior. If this revealed weight loss of ≥10 pounds, participants were asked whether this weight change was intentional. Unintentional weight loss was defined as answering “no” to this question. Serum creatinine was measured by a modified kinetic Jaffé reaction. Values from 1999–2000 were calibrated to the Cleveland Clinic laboratory standard by multiplying by 1.013 and then adding 0.147. Correction of values from 2001–2004 was not necessary. Estimated glomerular filtration rate (eGFR) was calculated using the Chronic Kidney Disease Epidemiology Collaboration (CKD-EPI) equation [20].

### Body composition

BMI was calculated using height and weight measured during the examination visit. Standing height was measured using a fixed stadiometer and weight using a calibrated digital scale. Whole-body DEXA scans were performed using a Hologic QDR-4500A fanbeam densitometer (Hologic, Inc., Bedford, Massachusetts). We quantified muscle mass using the appendicular skeletal muscle mass index (ASMI), calculated as total lean mass of the four extremities (by DEXA), divided by the square of the height [21]. We focused on appendicular muscle mass rather than total lean mass as the former is less confounded by differences in non-contractile lean mass. Low muscle mass was defined as ASMI <5.45 kg/m^2^ in women and <7.26 kg/m^2^ in men [21, 22]. This is a commonly used definition corresponding to two standard deviations below the sex-specific means for healthy young adults 18–40 years of age. This definition is recommended by the European Working Group on Sarcopenia in Older People to define low muscle mass for the classification of sarcopenia, and is consistent with the recommendations of other sarcopenia study groups [23, 24]. This enabled us to examine muscle mass and BMI independently, as opposed to the Foundation for the National Institutes of Health definition which includes BMI in the denominator. DEXA data were incomplete for at least one extremity in 1,914 participants (16.4%). Missing and invalid DEXA data were accounted for through multiple imputation by the National Center for Health Statistics [25]. Details of the DEXA quality control, data validation, and multiple imputation procedures are available [25–28]. Waist circumference was measured in 11,392 participants by drawing a horizontal line superior to the uppermost lateral border of the right ilium at the end of a normal expiration. Abdominal obesity was defined using National Cholesterol Education Program Adult Treatment Panel III cutpoints of 102 cm for men and 88 cm for women [29].

### All-cause mortality

Mortality status was ascertained through December 31, 2011 using public-use linked mortality files [30]. All-cause mortality was determined primarily through probabilistic record matching with the National Death Index [31]. Date and cause of death for selected records were subjected to data perturbation techniques due to concerns regarding participant anonymity, but vital status was not perturbed. The results of Cox proportional hazard models are not affected by these data perturbation techniques when compared with non-perturbed restricted-use data [32, 33].

### Statistical analysis

All analyses used NHANES-appropriate sampling weights and accounted for the complex multistage cluster design and multiple imputations using the survey estimation commands and ‘mi’ estimation suite in Stata version 13.1 (Stata Corporation, College Station, TX, USA). Participant characteristics were examined based on muscle mass status and then within categories of BMI stratified by muscle mass status. Differences between participants with low versus preserved muscle mass were tested for statistical significance using linear or logistic regression as appropriate. To determine if the relationship of total body fat percentage (%TBF) with BMI differed by muscle mass status, we examined scatterplots of %TBF with BMI and constructed b-splines including participants between the 1^st^ and 99^th^ percentiles of %TBF, by sex, to graphically model the potentially non-linear relationship. We also constructed linear splines to estimate clinically applicable parameters relating %TBF with BMI.

Cox proportional hazards regression models were created to test the hypothesis that muscle mass is a mediator of the relationship of BMI with mortality (S1 Fig). We first examined associations of BMI categories stratified by muscle mass status and then tested the effect of adding ASMI to a model containing BMI categories. BMI was categorized according to the World Health Organization definition, with the “normal” category divided at BMI = 22 kg/m^2^ based on the available sample size in our cohort and informed by prior literature indicating a nadir for risk of death around that number [1–6]. Models were adjusted *a priori* for potential confounders and included age, sex, race/ethnicity, smoking status, education level, and physical activity. We did not adjust for comorbidities as these may lie on the causal pathway between both BMI and muscle mass and mortality. The functional form of continuous variables was tested for linearity using higher-order terms and categorical variables. The proportional hazards and Weibull assumptions were verified by visual inspection of log-log survival curves. Causal mediation analyses were performed to further evaluate mediation effects of muscle mass (methods described in S1 Appendix) [34]. A p-value <0.05 was considered statistically significant.

## Results

### Participant characteristics

Overall 14.1% of participants had low muscle mass (Table 1). Compared with other participants, individuals with low muscle mass were older, less likely to be non-Hispanic black or never smokers, less active, more likely to have hypertension, CVD, or a history of cancer, and less likely to have diabetes. They also had lower BMI, %TBF, ASMI, and total lean mass. These patterns were similar when examined within BMI categories. However, within a BMI category, participants with low muscle mass had higher %TBF and waist circumference and were more likely to have diabetes and abdominal obesity compared with their counterparts with preserved muscle mass.

**Table 1:** Participant characteristics by muscle mass status and BMI in 11,687 participants of NHANES 1999–2004.

						Body Mass Index (kg/m^2^)			
				18.5 –<25		25 –<30		30–40	
Characteristic	Preserved Muscle Mass	Low Muscle Mass	P value	Preserved Muscle Mass	Low Muscle Mass	Preserved Muscle Mass	Low Muscle Mass	Preserved Muscle Mass	Low Muscle Mass
**Proportion (%)**	85.9 (0.5)	14.1 (0.5)		23.2 (0.7)	12.0 (0.4)	35.1 (0.7)	2.0 (0.2)	27.7 (0.7)	0.08 (0.02)
**Age (years)**	45.3 (0.3)	53.3 (0.4)	<0.001	40.0 (0.4)	51.3 (0.5)	47.0 (0.4)	64.4 (1.0)	47.6 (0.4)	70.7 (3.3)
**Women (%)** *	50.0 (0.5)	49.0 (1.5)	0.54	57.8 (1.0)	51.0 (1.6)	42.7 (1.0)	38.9 (4.3)	52.7 (1.0)	11.7 (10.4)
**Race/Ethnicity (%)**			<0.001						
*Non-Hispanic White*	71.2 (1.7)	77.8 (2.1)		72.5 (1.6)	77.2 (2.0)	70.6 (2.0)	81.1 (4.3)	70.9 (1.8)	85.7 (9.2)
*Mexican American*	7.4 (0.9)	6.7 (1.0)		6.0 (0.6)	6.7 (0.9)	8.1 (0.9)	6.7 (2.0)	7.8 (1.2)	6.8 (5.7)
*Non-Hispanic Black*	11.4 (1.0)	3.1 (0.4)		11.4 (1.1)	3.3 (0.4)	10.1 (1.1)	2.0 (0.8)	13.0 (1.1)	0
**BMI (kg/m**^**2**^**)**	28.2 (0.08)	22.4 (0.06)	<0.001	22.9 (0.04)	21.7 (0.05)	27.5 (0.03)	26.6 (0.07)	33.7 (0.05)	31.4 (0.7)
**Smoking (%)**			<0.001						
*Never*	50.7 (1.0)	43.0 (1.4)		52.8 (1.3	44.2 (1.6)	49.4 (1.2)	36.5 (3.5)	50.7 (1.4)	21.3 (12.6)
*Former*	25.0 (0.7)	27.4 (1.4)		18.4 (1.1)	25.4 (1.6)	27.3 (1.0)	38.4 (3.7)	27.4 (1.1)	64.4 (15.1)
*Current*	24.3 (0.7)	29.6 (1.8)		28.9 (1.2)	30.4 (2.1)	23.3 (0.9)	25.1 (3.5)	21.9 (1.0)	14.3 (10.4)
**Education (%)**			0.28						
*Did not graduate high school*	20.1 (0.7)	22.1 (1.7)		16.9 (1.0)	21.1 (1.6)	21.0 (1.2)	27.4 (4.1)	21.7 (0.8)	47.5 (16.3)
*High school graduate*	26.1 (0.7)	27.1 (1.4)		24.3 (1.1)	26.7 (1.3)	25.7 (1.0)	29.9 (3.9)	28.1 (1.1)	15.6 (10.3)
*Some college or AA degree*	29.5 (0.8)	27.4 (1.4)		28.8 (1.3)	27.8 (1.6)	29.1 (1.0)	26.5 (3.4)	30.5 (1.1)	0
*College graduate*	24.3 (1.2)	23.3 (1.8)		30.0 (1.9)	24.4 (2.0)	24.2 (1.3)	16.2 (3.1)	19.7 (1.0)	36.9 (16.5)
**Activity level (MET-min/wk, %)**			<0.001						
*0*	16.0 (0.7)	21.3 (1.1)		12.9 (0.8)	20.5 (1.2)	15.1 (1.0)	26.1 (3.2)	19.6 (0.7)	19.9 (13.5)
*<500*	20.2 (0.6)	22.6 (1.3)		17.4 (1.0)	22.1 (1.4)	20.2 (0.7)	25.1 (3.5)	22.5 (1.0)	35.1 (15.8)
*500–2000*	35.1 (0.6)	33.6 (1.5)		35.8 (1.2)	33.9 (1.5)	34.6 (0.9)	31.8 (4.2)	35.0 (1.1)	38.1 (14.8)
*>2000*	28.8 (0.7)	22.4 (1.3)		33.9 (1.3)	23.4 (1.5)	30.1 (1.0)	17.0 (2.5)	22.9 (0.9)	6.9 (7.7)
**Hypertension (%)**	39.7 (0.9)	44.0 (1.4)	0.007	22.6 (1.2)	39.5 (1.7)	40.2 (1.3)	69.8 (3.9)	53.3 (1.0)	85.9 (10.1)
**Diabetes mellitus (%)**	8.4 (0.4)	6.8 (0.7)	0.05	3.2 (0.4)	5.2 (0.8)	7.3 (0.5)	15.6 (2.4)	14.0 (0.7)	25.5 (13.7)
**Cardiovascular disease (%)**	7.9 (0.5)	13.3 (0.7)	<0.001	3.5 (0.5)	11.0 (0.7)	8.1 (0.5)	27.9 (3.5)	11.2 (0.7)	6.8 (7.6)
**History of cancer^ (%)**	6.0 (0.3)	11.7 (0.9)	<0.001	4.9 (0.5)	10.7 (0.8)	5.7 (0.5)	17.6 (2.7)	7.2 (0.5)	26.0 (14.1)
**% Total body fat**	33.9 (0.1)	32.2 (0.2)	<0.001	28.3 (0.2)	31.1 (0.2)	33.3 (0.2)	38.7 (0.6)	39.4 (0.2)	40.6 (1.3)
**Waist circumference (cm) (n = 11,392)**	96.6 (0.2)	85.6 (0.3)	<0.001	82.4 (0.2)	83.2 (0.3)	96.0 (0.2)	99.5 (0.7)	109.5 (0.2)	115.6 (2.1)
**Abdominal obesity (%) (n = 11,392)**	52.7 (1.0)	18.1 (1.4)	<0.001	7.3 (0.7)	9.3 (1.1)	49.0 (1.2)	69.5 (4.0)	95.8 (0.4)	100
**ASMI (kg/m**^**2**^**)**	7.79 (0.02)	5.94 (0.03)	<0.001	6.90 (0.03)	5.89 (0.03)	7.74 (0.03)	6.25 (0.08)	8.60 (0.03)	6.89 (0.19)
**Total lean mass (kg)**	51.5 (0.1)	41.3 (0.3)	<0.001	45.4 (0.2)	40.7 (0.3)	51.6 (0.2)	44.4 (0.8)	56.4 (0.2)	51.1 (0.2)

Abbreviations: NHANES, National Health and Nutrition Examination Survey; UACR, urine albumin-creatinine ratio; CRP, C-reactive protein; ASMI, appendicular skeletal muscle mass index.

Data are expressed as mean (standard error (SE)) or percent (SE).

* Proportion of women in BMI≥30, + sarcopenia category calculated using 3 out of the 5 imputations as in the other 2 there were no women in this category.

^ Excluding history of non-melanoma skin cancer

### Relationship of total body fat percentage with BMI

**Fig 1:**
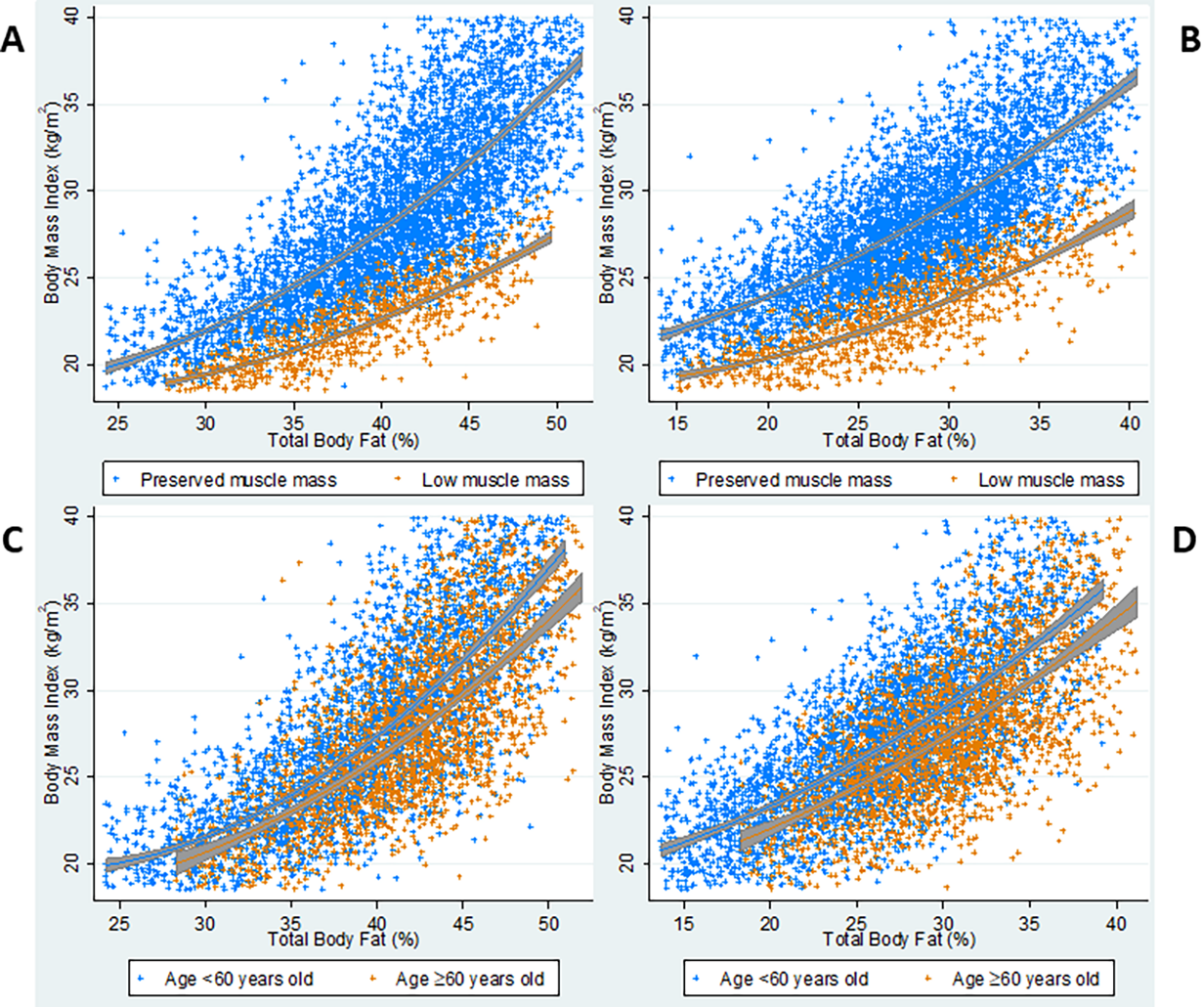
BMI versus total body fat percentage based on muscle mass status and age. Graphs show scatter plots of BMI and total body fat percentage and fitted b-splines based on muscle mass status for women (Panel A) and men (Panel B), and based on age <60 years or ≥60 years for women (Panel C) and men (Panel D). Scatter plots depict data for a single imputation but appeared identical for all imputations. B-splines were calculated using data from all imputations.

The presence of low muscle mass was not restricted to participants at the low end of the BMI or %TBF ranges. For both men and women, the association of %TBF with BMI differed markedly by muscle mass status (Fig 1A and 1B). At any level of %TBF, participants with low muscle mass had lower BMI than participants with preserved muscle mass. Visual inspection of b-splines demonstrated that this difference in BMI increased with higher %TBF. Between 30–40% TBF, 5% higher %TBF corresponded to a 2.6 kg/m^2^ (95% CI 2.4–2.8) higher BMI among women with preserved muscle mass, compared with a 1.5 kg/m^2^ (95% CI 1.3–1.7) difference among women with low muscle mass. In the 40–50% range, the difference in BMI per 5% higher TBF was 4.3 kg/m^2^ (95% CI 4.0–4.5) for women with preserved muscle mass compared with 2.4 kg/m^2^ (95% CI 2.2–2.7) for women with low muscle mass. The same pattern was present among men (S1 Table). Age did not account for the observed differences (Fig 1C and 1D).

### Associations of BMI with all-cause mortality

Over a median follow-up of 9.3 years (interquartile range 7.8–10.8), 1,819 participants died. Compared with participants with overweight BMI, the risk of death was similarly elevated among those with normal and obese BMI (Table 2). When separated by muscle mass status, within each BMI category the hazard ratio (HR) for death was higher for participants with low muscle mass (p<0.001 for interaction by muscle mass status). When restricted to individuals with preserved muscle mass, there was a significantly increased risk of death among those who were obese compared with overweight (HR 1.23, 95% CI 1.04–1.47), but not among those with BMI in the normal range (HR 1.09, 95% CI 0.90–1.31).

**Table 2:** Association of body-mass index stratified by muscle mass with all-cause mortality in 11,687 participants of NHANES 1999–2004.

		Hazard Ratio (95% CI)		
BMI (kg/m^2^)		Separated by muscle mass status		Relative to participants with preserved muscle mass in same BMI category
18.5 –<25 (n = 3,837)	**1.18 (1.04–1.35)**	Preserved muscle mass	1.09 (0.91–1.32)	
		Low muscle mass	**1.39 (1.19–1.63)**	**1.28 (1.07–1.52)**
25 –<30 (n = 4,503)	Reference	Preserved muscle mass	Reference	
		Low muscle mass	**1.50 (1.21–1.87)**	**1.52 (1.22–1.89)**
30–40 (n = 3,347)	1.18 (0.99–1.40)	Preserved muscle mass	**1.23 (1.03–1.47)**	
		Low muscle mass	**3.17 (1.48–6.77)**	**2.58 (1.20–5.53)**

Abbreviations: NHANES, National Health and Nutrition Examination Survey; BMI, body-mass index; CI, confidence interval

**Bold** denotes p<0.05.

**Fig 2:**
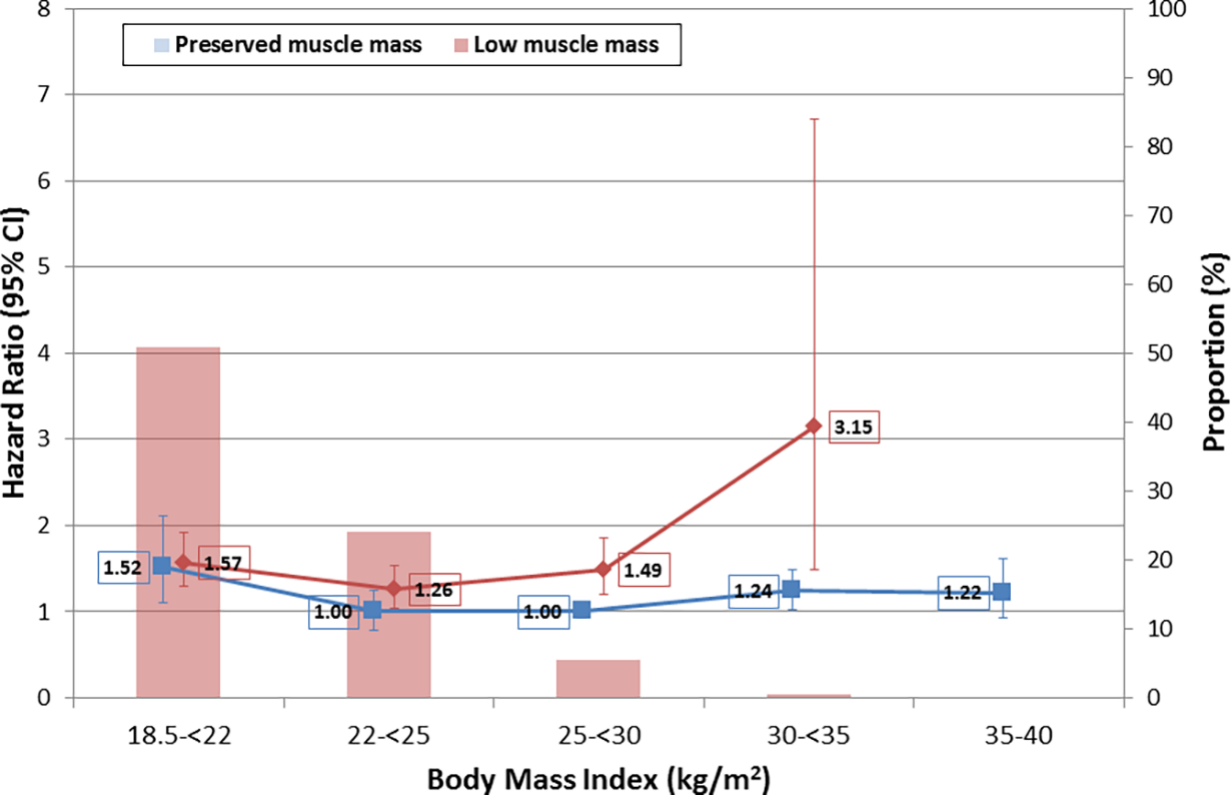
Risk of all-cause mortality by BMI category and muscle mass status. Bars indicate prevalence of low muscle mass in each BMI category. One participant with BMI >35 (38 kg/m^2^) had low muscle mass and was grouped with participants with BMI 30-<35 kg/m^2^ for statistical analysis. Models adjusted for age, sex, race/ethnicity, smoking status, physical activity level, and education. Error bars represent 95% confidence intervals.

**Fig 3:**
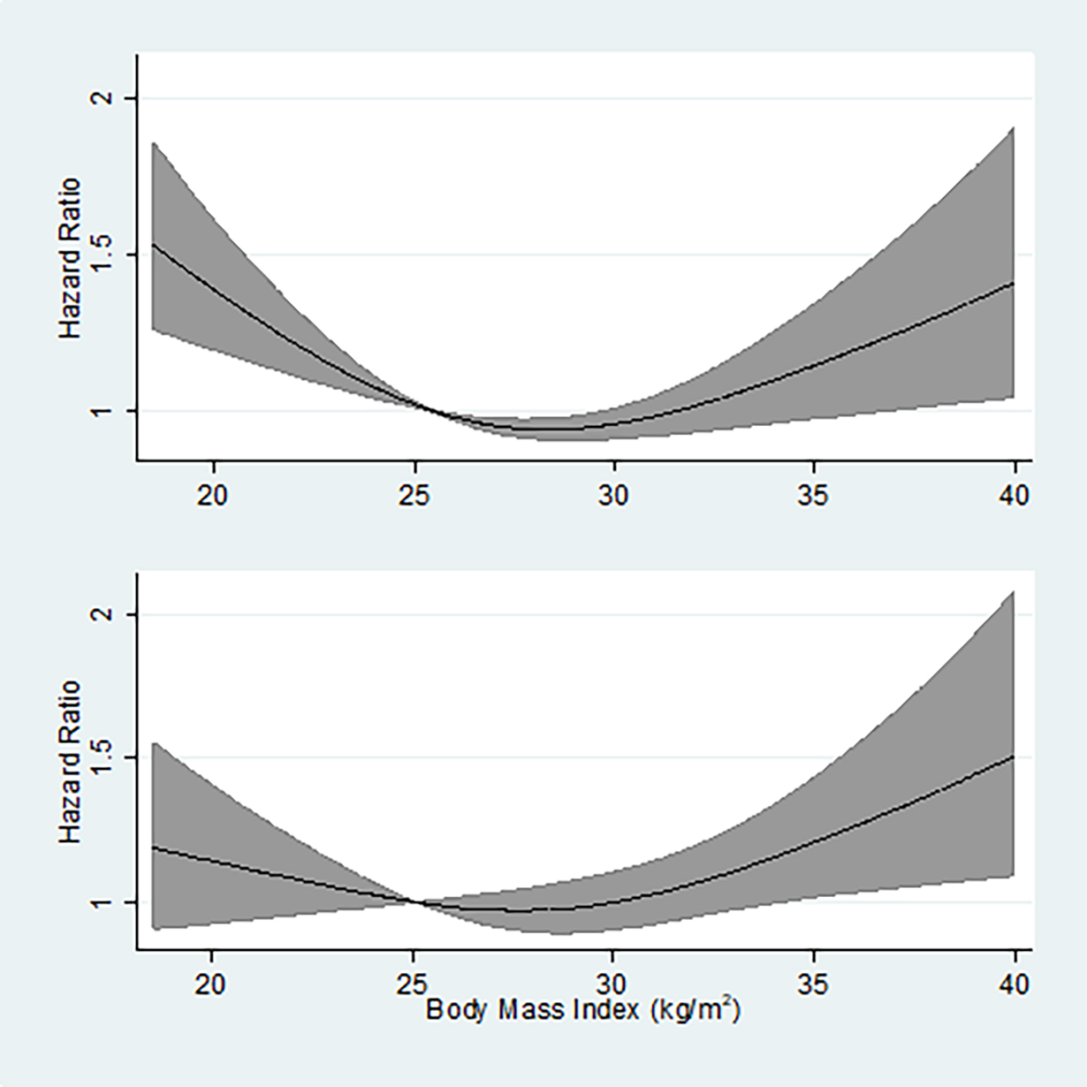
The risk of death according to BMI for the full cohort (upper panel) and for participants with preserved muscle mass (lower panel). Mortality modeled as a restricted cubic spline and models adjusted for age, sex, race/ethnicity, smoking status, physical activity level, and education. The shaded area represents the 95% confidence interval.

After further subdividing the normal and obese BMI categories, a U-shaped association was apparent for participants with both low and preserved muscle mass. The risk of death was greater for those with low muscle mass in each BMI category other than 18.5-<22 kg/m^2^ (Fig 2). However, the prevalence of low muscle mass decreased precipitously with increasing BMI (Fig 2; 24.1%, 5.4%, and 0.5% for 22-<25, 25-<30, and ≥30 kg/m^2^, respectively). The difference in prevalence across BMI categories suggested that combining participants irrespective of muscle mass would increase the HR more for BMI levels in the low-normal range than for levels in the overweight or obese range. Restricted cubic splines in the full cohort compared with participants with preserved muscle mass were consistent with this hypothesis, as in the latter the increased risk with low BMI and the apparent protective association of overweight BMI were both attenuated (Fig 3, upper and lower panels, respectively). These results were similar for men and women (S2 and S3 Figs).

**Fig 4:**
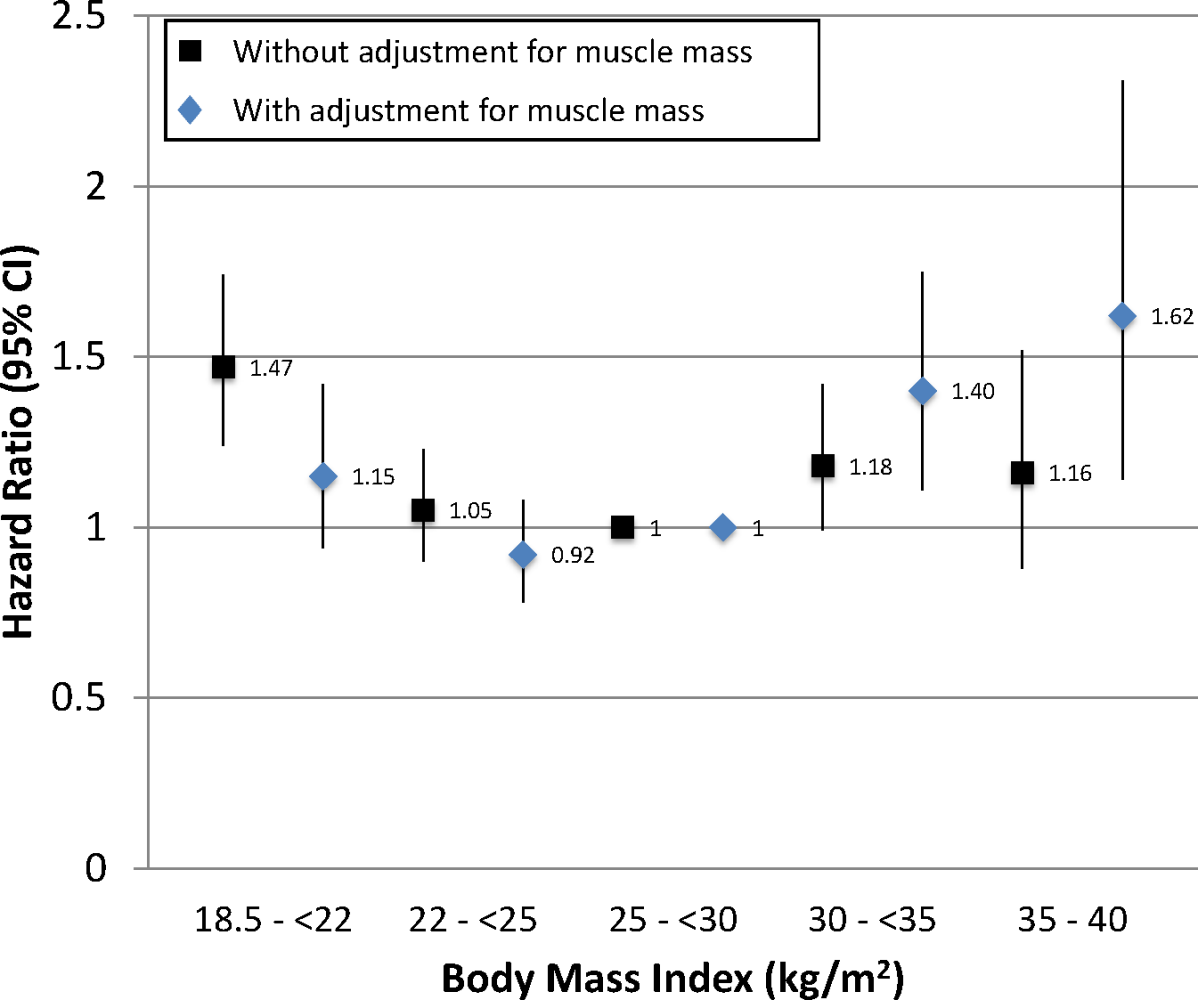
Association of BMI with all-cause mortality without and with adjustment for appendicular skeletal muscle mass index for the full cohort. Models adjusted for age, sex, race/ethnicity, smoking status, physical activity level, and education. Error bars represent 95% confidence intervals.

**Fig 5:**
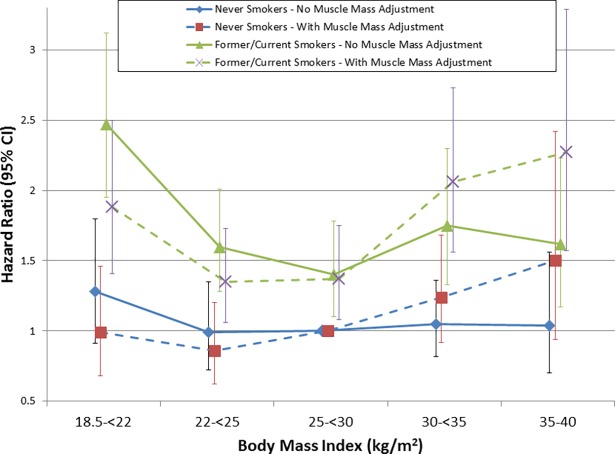
Association of BMI with all-cause mortality without and with adjustment for appendicular skeletal muscle mass index after stratification by smoking status. Models adjusted for age, sex, race/ethnicity, smoking status, physical activity level, and education. Error bars represent 95% confidence intervals.

We next examined whether a similar effect on mortality was present without categorizing individuals based on a threshold value of muscle mass. Compared with a BMI of 25-<30 kg/m^2^, adjusting for ASMI as a continuous variable tended to reduce the risk of death associated with BMI <25 kg/m^2^ and to magnify the risk associated with BMI ≥30 kg/m^2^, and suggested that the lowest risk of death was associated with BMI in the 22–25 kg/m^2^ range (Fig 4). This effect was more prominent among Americans under 60 years of age, but in those ≥60 years old there was still a trend towards a relative increase in the risk of death for BMI ≥25 kg/m^2^ (S4 and S5 Figs). Causal mediation analyses supported that muscle mass partially mediated the association of BMI with mortality, both in younger and older Americans (S1 Appendix). Higher ASMI was independently associated with a reduced risk of death (HR 0.82 per 1 kg/m^2^ (95% CI 0.73–0.92)). This also was of greater magnitude among younger Americans but remained significant among older Americans (S2 Table). Adjustment for ASMI in models stratified by smoking status produced a similar effect on BMI-associated mortality (Fig 5), indicating that muscle mass provided additional prognostic information even when smokers and non-smokers were examined separately.

**Fig 6:**
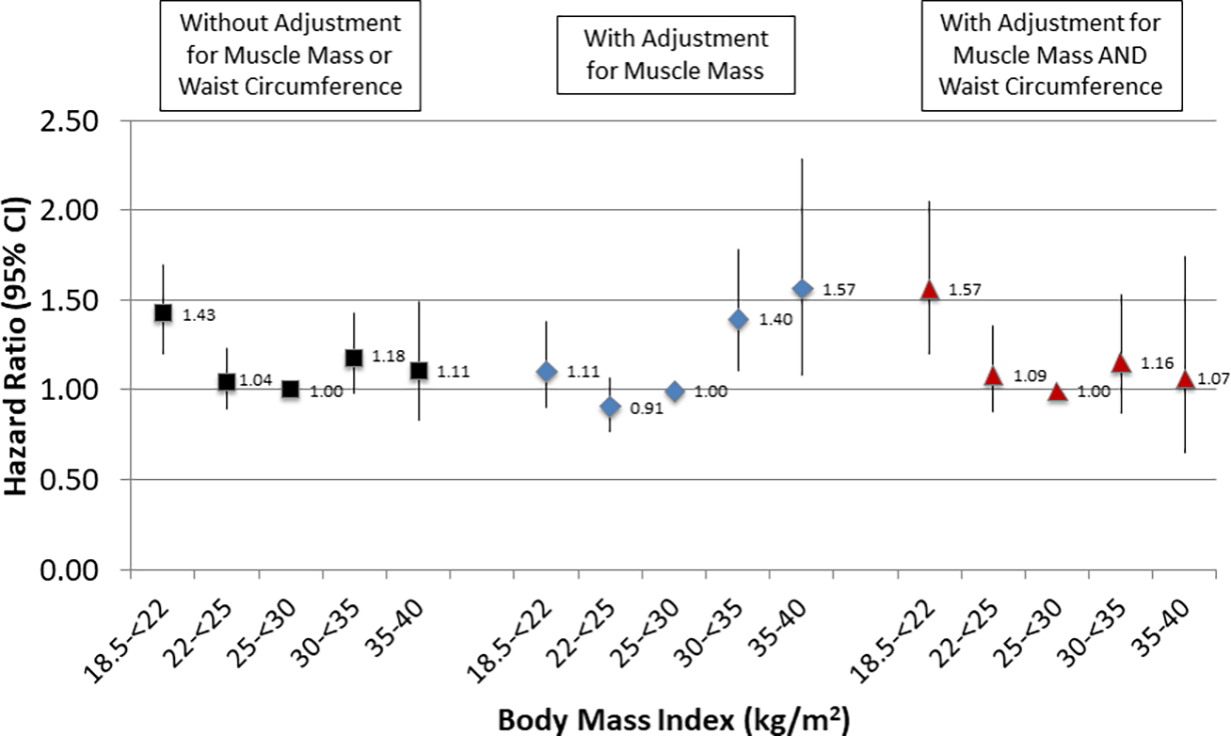
Association of BMI with all-cause mortality without and with adjustment for appendicular skeletal muscle mass index and waist circumference. Models adjusted for age, sex, race/ethnicity, smoking status, physical activity level, and education. Error bars represent 95% confidence intervals.

**Fig 7:**
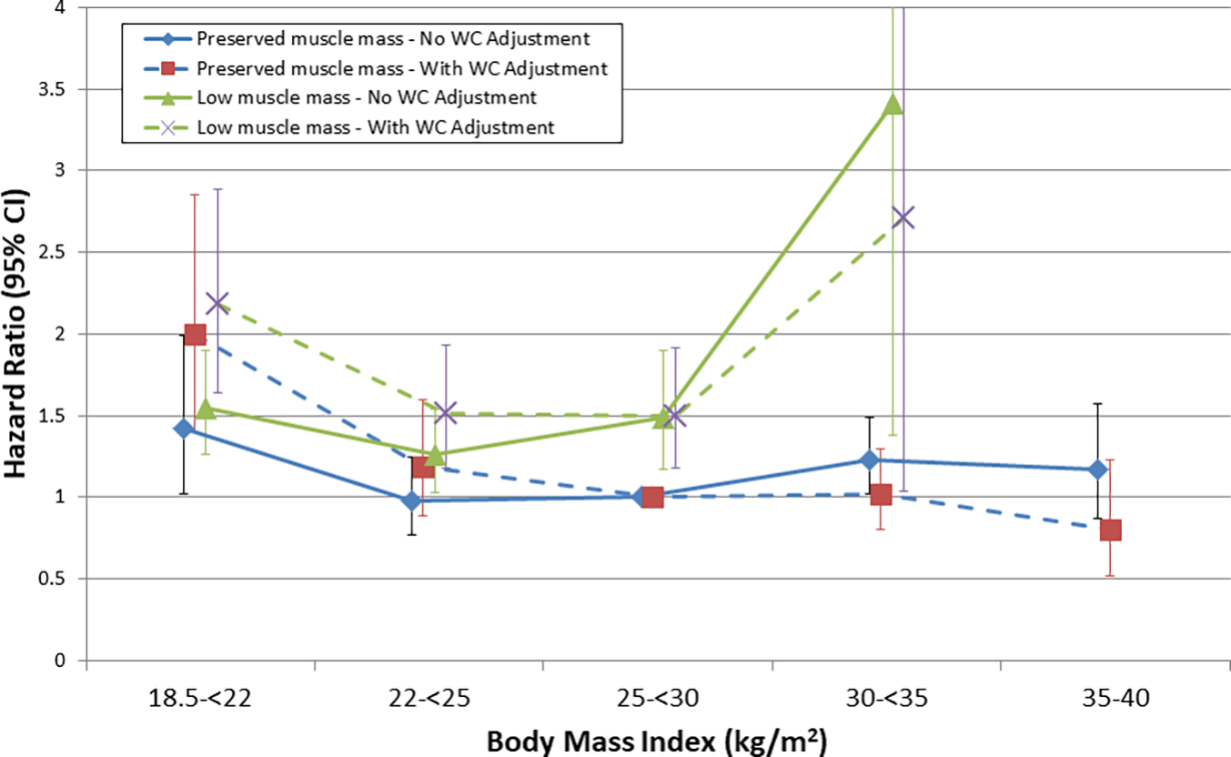
Risk of mortality by BMI category and muscle mass status, without and with adjustment for waist circumference. Y-axis truncated for clarity (see S6 Fig for untruncated y-axis). Models adjusted for age, sex, race/ethnicity, smoking status, physical activity level, and education. Error bars represent 95% confidence intervals.

We hypothesized that after accounting for differences in muscle mass, higher BMI would specifically capture increased adiposity. Therefore, the increased mortality we observed would be attenuated by adjustment for waist circumference. Indeed, adding waist circumference to the model in addition to ASMI eliminated the risk associated with higher BMI (Fig 6). We also considered the possibility that greater abdominal obesity (Table 1) could explain the effect of low muscle mass on the BMI-mortality association. After stratification by muscle mass status, adjustment for waist circumference attenuated the risk of death for higher BMI but did not reduce effect modification by low muscle mass (Fig 7, S6 Fig). Rather, higher BMI was then associated with reduced mortality among participants with preserved muscle mass (HR 0.95 per kg/m^2^ (95% CI 0.91–0.98)). Further, the hazard ratio for ASMI did not change after adding waist circumference to the model (HR 0.81 (95% CI 0.72–0.92); S2 Table).

#### Sensitivity analyses

We conducted several analyses to determine the effects of chronic illness and subclinical or undiagnosed disease on our estimates. Excluding individuals with unintentional weight loss in the prior 12 months; participants with diabetes, congestive heart filure, a history of cancer, or eGFR<30 mL/min/1.73m^2^; or those who died within the first 2 years of follow-up, did not change our findings (S7–S12 Figs, S2 Table). Results were similar when restricting analyses to participants who reported not performing muscle strengthening activities or who did not meet minimum weekly physical activity recommendations (≥500 MET-min/wk) (S2 Table), demonstrating that our findings were not driven by greater muscle mass among people participating in exercise or regular physical activity regimens. Results were also not different when adjusting for the poverty-income ratio as an additional measure of socioeconomic status in the subgroup who reported this information (S2 Table).

## Discussion

Our results from a nationally representative cohort demonstrate that skeletal muscle mass is an important mediator and effect modifier of the relationship of BMI with mortality. Both the exclusion of participants with low muscle mass and adjustment for muscle mass as a continuous variable attenuated the risk associated with low BMI, magnified the risk associated with high BMI, and shifted downward the level of BMI associated with the lowest risk of death.

We found that muscle mass alters the relationship of BMI with body fat percentage and with the sequelae of excess body fat. Although overall people with low muscle mass had less body fat and a lower BMI than people with preserved muscle mass, at a given level of BMI those with low muscle mass had higher %TBF and waist circumference, were more likely to have diabetes, and had an increased risk of death. Furthermore, large differences in body fat in these individuals manifested as relatively small differences in BMI. Therefore, using BMI as the sole measure of body composition inappropriately lumps together people with different degrees of adiposity and disparate risks of death.

Some have hypothesized that excess fat stores are beneficial for counteracting episodes of catabolic stress [9–11]. If so, people with low muscle mass should benefit most from the energy reserves provided by excess adiposity. However, the risk of death associated with low muscle mass was not reduced by greater body fat as represented by higher BMI, but rather increased. Thus, our data are not consistent with a survival advantage related to overweight or obesity.

Our findings extend those of prior cohort studies that associated the lowest risk of mortality with a BMI between 20 and 24.9 [1–6]. In all models adjusting for ASMI, the HR for BMI 22–24.9 changed from >1 before adjustment to <1 after adjustment, and the risk associated with BMI <22 was substantially attenuated. This finding was unchanged in analyses accounting for the possible confounding effects of chronic illness. Furthermore, a similar effect was observed in analyses restricted to never smokers as in analyses that were not stratified by smoking status. Of note, 57% of people with low muscle mass had never smoked and many did not have comorbidities associated with muscle wasting. Therefore, restricting analyses to never smokers or people without chronic disease, as others have done [1, 2, 4–6], does not eliminate clinically meaningful variability in muscle mass that limits the utility of BMI. Our definition of low muscle mass did not drive these results, as analyses modeling ASMI as a continuous variable led to the same conclusion. Taken together, these data indicate that if one is to draw conclusions regarding the risks of overweight and obesity by examining the association of BMI with mortality, failure to account for differences in muscle mass inappropriately minimizes those risks.

Even analyses using direct measures of adiposity likely underestimate the risks of excess body fat as they fail to account for the increases in muscle mass that typically accompany obesity. These changes in muscle impact mortality risk independently of, and in a direction opposite to, the effect of increased body fat.

We found that appendicular skeletal muscle mass was an independent risk factor for mortality in the general population, and this was more pronounced among younger Americans. This is striking given that the risks of low muscle mass have been a major focus in the geriatric literature and in cohorts with various chronic diseases, but not otherwise in the general population [35–37]. Due to the observational nature of this study, we are unable to determine whether this is a causal relationship. There may be residual confounding due to undiagnosed disease or other health-related factors. Alternatively, skeletal muscle mass could directly influence survival. Greater muscle mass could protect against loss of functional status due to aging or the onset of chronic disease. The protein stores provided by muscle could be beneficial during episodes of catabolic stress. Skeletal muscle may also regulate whole-body metabolism and inflammation [38, 39]. Future studies will be needed to further evaluate this finding.

These results are based on a single assessment of body composition, which limits our ability to address the cause of low muscle mass or to incorporate temporal changes. For example, weight regain after weight loss in older persons leads to relatively more fat accumulation and less muscle gain than was lost [40, 41]. As a result, weight cycling could lead to lower muscle mass than expected for a given level of BMI, which could partly account for the association of high body weight variability with mortality [42]. Due to the small sample size, we were also unable to examine the effect of race and ethnicity on our findings. This could be important to explore in the future, as blacks were more likely than whites to have preserved muscle mass, and several studies have observed less risk associated with high BMI in blacks compared with whites [5, 43–45].

Other limitations should also be considered. Our analyses included data that were imputed by the National Center for Health Statistics; however, the resultant scatterplots of %TBF with BMI were strikingly similar to prior reports with complete DEXA data [46, 47]. Although inclusion of ASMI, waist circumference, and BMI in the same model might raise concerns about multicollinearity, estimates for ASMI and waist circumference were the same when examined separately or together.

### Conclusions

Skeletal muscle mass modifies the association of BMI with adiposity and with mortality. After accounting for muscle mass, the level of BMI associated with the greatest survival shifts downward toward the normal range.

## Supporting information

S1 Appendix Causal mediation analysis. (DOCX)

S1 Table Difference in body-mass index per 5% higher total body fat percentage. (DOCX)

S2 Table Associations with all-cause mortality in the full cohort and in sensitivity analyses. (DOCX)

S1 Fig Causal diagram depicting relationship of BMI, muscle mass, and mortality. (PPTX)

S2 Fig Association of BMI with all-cause mortality modeled as a restricted cubic spline for all men (upper panel) and for men with preserved muscle mass (lower panel). The shaded area represents the 95% confidence interval.
(DOCX)

S3 Fig Association of BMI with all-cause mortality modeled as a restricted cubic spline for all women (upper panel) and for women with preserved muscle mass (lower panel). The shaded area represents the 95% confidence interval.
(DOCX)

S4 Fig Association of BMI with all-cause mortality without and with adjustment for appendicular skeletal muscle mass index among Americans younger than 60 years of age (n = 7,395). Error bars represent 95% confidence intervals.
(DOCX)

S5 Fig Association of BMI with all-cause mortality without and with adjustment for appendicular skeletal muscle mass index among Americans 60 years of age and older (n = 4,292). Error bars represent 95% confidence intervals.
(DOCX)

S6 Fig Risk of all-cause mortality by BMI category and muscle mass status, without and with adjustment for waist circumference (n = 11,392). Error bars represent 95% confidence intervals.
(DOCX)

S7 Fig Risk of mortality by BMI category and muscle mass status among participants who did not report unintentional weight loss in the previous 12 months (n = 10,867). Bars indicate prevalence of low muscle mass in each BMI category.
(DOCX)

S8 Fig Association of BMI with all-cause mortality without and with adjustment for appendicular skeletal muscle mass index among participants who did not report unintentional weight loss in the previous 12 months (n = 10,867). Error bars represent 95% confidence intervals.
(DOCX)

S9 Fig Risk of mortality by BMI category and muscle mass status after excluding participants with a diagnosis of diabetes mellitus, congestive heart failure, a history cancer (other than non-melanoma skin cancer), or an estimated glomerular filtration rate <30 mL/min/1.73m^2^ (n = 8,802). Bars indicate prevalence of low muscle mass in each BMI category.
(DOCX)

S10 Fig Association of BMI with all-cause mortality without and with adjustment for appendicular skeletal muscle mass index after excluding participants with a diagnosis of diabetes mellitus, congestive heart failure, a history cancer (other than non-melanoma skin cancer), or an estimated glomerular filtration rate <30 mL/min/1.73m^2^ (n = 8,802). Error bars represent 95% confidence intervals.
(DOCX)

S11 Fig Risk of mortality by BMI category and muscle mass status after excluding participants who died within the first 2 years of follow-up (n = 11,395). Bars indicate prevalence of low muscle mass in each BMI category.
(DOCX)

S12 Fig Association of BMI with all-cause mortality without and with adjustment for appendicular skeletal muscle mass index after excluding participants who died within the first 2 years of follow-up (n = 11,395). Error bars represent 95% confidence intervals.
(DOCX)
