# Correction: Muscle mass, BMI, and mortality among adults in the United States: A population-based cohort study

**DOI:** 10.1371/journal.pone.0198318

**Published:** 2018-05-24

**Authors:** Matthew K. Abramowitz, Charles B. Hall, Afolarin Amodu, Deep Sharma, Lagu Androga, Meredith Hawkins

There are errors in the Funding section. The correct funding information is as follows: This research was supported by K23 DK099438 to MKA and R01 DK069861 and R01 DK079974 to MH from the National Institute of Diabetes and Digestive and Kidney Diseases of the National Institutes of Health (https://www.niddk.nih.gov), and by the Einstein-Montefiore NIH CTSA Grant UL1TR001073 from the National Center for Research Resources (NCRR) and the Einstein-Mt. Sinai Diabetes Research Center (5P30DK020541-41). The funder had no role in study design, data collection and analysis, decision to publish, or preparation of the manuscript.

There are errors in the Acknowledgments section. The Acknowledgments should read: This research was supported by K23 DK099438 from the National Institutes of Health (NIH). This work was also supported by grants from the NIH (DK069861 and DK079974), by the Einstein-Montefiore NIH CTSA Grant UL1TR001073 from the National Center for Research Resources (NCRR) and the Einstein-Mt. Sinai Diabetes Research Center (5P30DK020541-41). Its contents are solely the responsibility of the authors and do not necessarily represent the official views or policies of the NCRR or NIH.
