# Supplementary material for: Muscle mass, BMI, and mortality among adults in the United States: A population-based cohort study
Source: PLoS One. 2018 Apr 11;13(4):e0194697. doi: 10.1371/journal.pone.0194697 (PMC5894968; doi:10.1371/journal.pone.0194697)
Supplement: S1 Appendix — (DOCX) [file pone.0194697.s001.docx]

**S1 Appendix. Causal mediation analysis**

**Methods**. Mediation may be measured by the difference in effects between a model that includes the mediator (regression direct effect) and one that does not include the mediator (total regression effect). This requires no interaction between the exposure and the mediator, which may not be the case here. Therefore we performed a causal mediation analysis similar to that of VanderWeele (48), modeling survival using an accelerated failure time model with a Weibull distribution. The assumptions for a causal mediation analysis with a proportional hazards model are only met if the outcome is rare (49). We fit the following models:

$$\log T_{i}=\theta_{0}+\theta_{1}a_{1i}+\theta_{2}a_{2i}+\theta_{3}a_{3i}+\theta_{4}a_{4i}+\theta_{m}m_{i}+\theta_{int1}a_{1i}m_{i}+\theta_{int2}a_{2i}m_{i}+\theta_{int3}a_{3i}m_{i}+\theta_{int4}a_{4i}m_{i}+\theta_{c}^{'}c_{i}+\sigma\varepsilon_{i}$$

For low muscle mass as the mediator:

$$log\left\{ \frac{Pr\left( \left\{ m_{i}=1 | a_{1i},a_{2i},a_{3i},a_{4i},c_{i} \right\} \right)}{1-Pr\left( \left\{ m_{i}=1 | a_{1i},a_{2i},a_{3i},a_{4i},c_{i} \right\} \right)} \right\}=\beta_{0}+\beta_{1}a_{1i}+\beta_{2}a_{2i}+\beta_{3}a_{3i}+\beta_{4}a_{4i}+\beta_{c}^{'}c_{i}$$

For the appendicular skeletal muscle mass index (ASMI) as the mediator:

$$m_{i}=\beta_{0}+\beta_{1}a_{1i}+\beta_{2}a_{2i}+\beta_{3}a_{3i}+\beta_{4}a_{4i}+\beta_{c}^{'}c_{i}$$

where T_i_ is the follow-up time; ε_i_ is the residual variation in the log-transformed survival times; σ is a scale parameter; a_1i_ – a_4i_ are dummy variables, each taking the value of 1 for a BMI category (18.5 - <22, 22 - <25, 30 - <35, and 35-40 kg/m^2^, respectively) and 0 otherwise, with 25 - <30 kg/m^2^ as the reference category; m_i_ is the mediator; and c_i_ is a vector containing all other covariate values. When we examined mediation by low muscle mass, because of the small number of participants with obese BMI and low muscle mass, the two highest BMI categories were combined and only a_1i_ – a_3i_ were used for dummy variables.

For BMI 18.5 - <22 kg/m^2^, a_1i_, we estimated the following (50):

CDE_1_ = θ_1_

INT_ref1_ = θ_int1_(β_0_ + β’_c_c_i_)a_1i_

INT_med1_ = θ_int1_β_1_

PIE_1_ = θ_m_β_1_

TE_1_ = CDE_1_ + INT_ref1_ + INT_med1_ + PIE_1_,

where the controlled direct effect (CDE) is the effect of BMI on all-cause mortality when the mediator is not present; INT_ref_ is the reference interaction between BMI and other predictors not including the mediator; INT_med_ is the interaction between BMI and the mediator; PIE is the pure indirect effect on mortality from the product effect of BMI through the mediator; and TE is the total effect of BMI on mortality. We similarly estimated the above parameters for the remaining BMI categories. INT_ref_ was estimated using the following covariate values: 51 year old (the mean age) non-Hispanic white woman, never smoker, without a high-school diploma, and reporting the lowest level of physical activity.

The appendicular skeletal muscle mass index was entered into the model as the sex-specific arithmetic distance from the low muscle mass threshold, as defined in the main text of the paper: ASMI - 5.45 for women; ASMI - 7.26 for men. We also performed causal mediation analyses for the appendicular skeletal muscle mass index in participants <60 and ≥60 years old, separately.

**Results**. There was a significant mediated effect (PIE) through muscle mass at all levels of BMI using either low muscle mass or the appendicular skeletal muscle mass index as the mediator (see Tables below). Significant mediated effects were also noted in both age subgroups. Interaction effects were not statistically significant.

| **Causal mediation analysis examining low muscle mass as a mediator of the association of BMI with all-cause mortality** | | | | | | |
| --- | --- | --- | --- | --- | --- | --- |
|  | Body Mass Index (kg/m^2^) | | | | | |
|  | 18.5-<22 | | 22-<25 | | 30-40 | |
|  | Estimate (95% CI) | p | Estimate (95% CI) | p | Estimate (95% CI) | p |
| **Causal mediation models** |  |  |  |  |  |  |
| Controlled direct effect | -0.29 (-0.51 to -0.07) | 0.01 | 0 (-.15 to .15) | 0.99 | -0.14 (-0.27 to -0.02) | 0.02 |
| Interaction in absence of mediator | -0.86 (-1.89 to 0.17) | 0.10 | -0.37 (-1.20 to 0.45) | 0.37 | 1.21 (-0.59 to 3.00) | 0.18 |
| Mediated interaction | 0.99 (-0.17 to 2.16) | 0.09 | 0.24 (-0.29 to 0.77) | 0.36 | 1.07 (-0.53 to 2.66) | 0.18 |
| Pure indirect effect | -1.05 (-1.63 to -0.47) | <0.001 | -0.59 (-0.91 to -0.28) | <0.001 | 0.80 (0.33 to 1.27) | 0.001 |
| Total effect | -1.21 (-1.78 to -0.63) | <0.001 | -0.72 (-1.38 to -0.06) | 0.03 | 2.93 (-0.42 to 6.29) | 0.08 |
|  |  |  |  |  |  |  |
| **Results from conventional models** |  |  |  |  |  |  |
| Regression direct effect | -0.14 (-0.26 to -0.02) | 0.03 | 0.03 (-0.08 to 0.14) | 0.55 | -0.13 (-0.26 to -0.01) | 0.03 |
| Total regression effect | -0.26 (-0.38 to -0.15) | <0.001 | -0.04 (-0.15 to 0.07) | 0.48 | -0.11 (-0.23 to 0.01) | 0.06 |

| **Causal mediation analysis examining the appendicular skeletal muscle mass index as a mediator of the association of BMI with all-cause mortality** | | | | | | | | |
| --- | --- | --- | --- | --- | --- | --- | --- | --- |
|  | Body Mass Index (kg/m^2^) | | | | | | | |
|  | 18.5-<22 | | 22-<25 | | 30-<35 | | 35-40 | |
|  | Estimate (95% CI) | p | Estimate (95% CI) | p | Estimate (95% CI) | p | Estimate (95% CI) | p |
| **Causal mediation models** |  |  |  |  |  |  |  |  |
| Controlled direct effect | -0.08 (-0.23 to 0.08) | 0.31 | 0.09 (-0.01 to 0.19) | 0.08 | -0.10 (-0.34 to 0.15) | 0.42 | -0.01 (-0.59 to 0.57) | 0.96 |
| Interaction in absence of mediator | -0.07 (-0.25 to 0.10) | 0.40 | 0.03 (-0.15 to 0.22) | 0.71 | -0.11 (-0.25 to 0.03) | 0.13 | -0.16 (-0.36 to 0.05) | 0.12 |
| Mediated interaction | 0.10 (-0.13 to 0.33) | 0.40 | -0.03 (-0.17 to 0.11) | 0.71 | -0.09 (-0.22 to 0.03) | 0.13 | -0.28 (-0.64 to 0.08) | 0.13 |
| Pure indirect effect | -0.24 (-0.37 to -0.10) | 0.001 | -0.14 (-0.22 to -0.06) | 0.001 | 0.16 (0.07 to 0.25) | 0.001 | 0.32 (0.14 to 0.50) | 0.001 |
| Total effect | -0.29 (-0.42 to -0.17) | <0.001 | -0.04 (-0.17 to 0.09) | 0.54 | -0.14 (-0.27 to -0.01) | 0.04 | -0.13 (-0.32 to 0.06) | 0.17 |
|  |  |  |  |  |  |  |  |  |
| **Results from conventional models** |  |  |  |  |  |  |  |  |
| Regression direct effect | -0.10 (-0.24 to 0.04) | 0.16 | 0.05 (-0.05 to 0.16) | 0.31 | -0.23 (-0.39 to -0.07) | 0.006 | -0.33 (-0.57 to -0.09) | 0.008 |
| Total regression effect | -0.26 (-0.38 to -0.15) | <0.001 | -0.04 (-0.15 to 0.07) | 0.48 | -0.12 (-0.24 to 0.01) | 0.07 | -0.10 (-0.29 to 0.08) | 0.27 |

| **Age-stratified causal mediation analysis examining the appendicular skeletal muscle mass index as a mediator of the association of BMI with all-cause mortality** | | | | | | | | |
| --- | --- | --- | --- | --- | --- | --- | --- | --- |
|  | Body Mass Index (kg/m^2^) | | | | | | | |
|  | 18.5-<22 | | 22-<25 | | 30-<35 | | 35-40 | |
|  | Estimate (95% CI) | p | Estimate (95% CI) | p | Estimate (95% CI) | p | Estimate (95% CI) | p |
| *Age <60 (n=7,395)* |  |  |  |  |  |  |  |  |
| **Causal mediation models** |  |  |  |  |  |  |  |  |
| Controlled direct effect | 0.10 (-0.28 to 0.49) | 0.59 | 0.25 (-0.09 to 0.58) | 0.14 | -0.34 (-1.09 to 0.40) | 0.36 | -1.47 (-2.80 to -0.14) | 0.03 |
| Interaction in absence of mediator | -0.28 (-0.79 to 0.24) | 0.29 | 0.03 (-0.57 to 0.63) | 0.92 | -0.19 (-0.65 to 0.26) | 0.40 | 0.16 (-0.51 to 0.84) | 0.62 |
| Mediated interaction | 0.31 (-0.26 to 0.89) | 0.28 | -0.02 (-0.41 to 0.37) | 0.92 | -0.15 (-0.49 to 0.20) | 0.40 | 0.24 (-0.75 to 1.23) | 0.62 |
| Pure indirect effect | -0.52 (-0.92 to -0.12) | 0.01 | -0.30 (-0.54 to -0.07) | 0.01 | 0.35 (0.08 to 0.62) | 0.01 | 0.69 (0.17 to 1.20) | 0.01 |
| Total effect | -0.38 (-0.76 to 0.00) | 0.05 | -0.05 (-0.40 to 0.31) | 0.80 | -0.33 (-0.63 to -0.03) | 0.03 | -0.38 (-0.87 to 0.12) | 0.13 |
|  |  |  |  |  |  |  |  |  |
| **Results from conventional models** |  |  |  |  |  |  |  |  |
| Regression direct effect | 0.10 (-0.29 to 0.48) | 0.62 | 0.22 (-0.06 to 0.50) | 0.12 | -0.62 (-1.02 to -0.23) | 0.003 | -1.02 (-1.63 to -0.41) | 0.002 |
| Total regression effect | -0.38 (-0.75 to -0.02) | 0.04 | -0.06 (-0.39 to 0.27) | 0.74 | -0.32 (-0.60 to -0.03) | 0.03 | -0.42 (-0.87 to 0.02) | 0.06 |
|  |  |  |  |  |  |  |  |  |
| *Age ≥60 (n=4,292)* |  |  |  |  |  |  |  |  |
| **Causal mediation models** |  |  |  |  |  |  |  |  |
| Controlled direct effect | -0.10 (-0.28 to 0.08) | 0.27 | 0.06 (-0.06 to 0.19) | 0.32 | -0.11 (-0.37 to 0.15) | 0.41 | 0.42 (-0.13 to 0.96) | 0.13 |
| Interaction in absence of mediator | 0.01 (-0.11 to 0.14) | 0.83 | 0.03 (-0.10 to 0.16) | 0.64 | -0.02 (-0.12 to 0.08) | 0.72 | -0.17 (-0.32 to -0.02) | 0.03 |
| Mediated interaction | -0.02 (-0.25 to 0.20) | 0.83 | -0.03 (-0.16 to 0.10) | 0.64 | -0.02 (-0.14 to 0.10) | 0.72 | -0.44 (-0.83 to -0.05) | 0.03 |
| Pure indirect effect | -0.12 (-0.22 to -0.01) | 0.04 | -0.07 (-0.13 to -0.01) | 0.03 | 0.08 (0.01 to 0.15) | 0.04 | 0.17 (0.01 to 0.33) | 0.04 |
| Total effect | -0.22 (-0.35 to -0.10) | <0.001 | 0 (-0.12 to 0.11) | 0.94 | -0.07 (-0.18 to 0.05) | 0.24 | -0.02 (-0.17 to 0.13) | 0.79 |
|  |  |  |  |  |  |  |  |  |
| **Results from conventional models** |  |  |  |  |  |  |  |  |
| Regression direct effect | -0.14 (-0.27 to -0.01) | 0.04 | 0.04 (-0.09 to 0.17) | 0.54 | -0.12 (-0.27 to 0.02) | 0.08 | -0.16 (-0.36 to 0.03) | 0.10 |
| Total regression effect | -0.22 (-0.34 to -0.10) | 0.001 | -0.01 (-0.12 to 0.11) | 0.89 | -0.06 (-0.18 to 0.05) | 0.28 | -0.04 (-0.18 to 0.11) | 0.63 |
