# Supplementary material for: Muscle mass, BMI, and mortality among adults in the United States: A population-based cohort study
Source: PLoS One. 2018 Apr 11;13(4):e0194697. doi: 10.1371/journal.pone.0194697 (PMC5894968; doi:10.1371/journal.pone.0194697)
Supplement: S1 Table — (DOCX) [file pone.0194697.s002.docx]

| **S1 Table. Difference in body-mass index per 5% higher total body fat percentage** | | | |
| --- | --- | --- | --- |
| Total body fat percentage | Preserved muscle mass | Low muscle mass | P for interaction by muscle mass status |
| Women |  |  | <0.001 |
| 30-40% | 2.6 (2.4-2.8) | 1.5 (1.3-1.7) |  |
| 40-50% | 4.3 (4.0-4.5) | 2.4 (2.2-2.7) |  |
|  |  |  |  |
| Men |  |  | <0.001 |
| 20-30% | 2.7 (2.5-2.9) | 1.7 (1.5-2.0) |  |
| 30-40% | 3.5 (3.2-3.8) | 2.5 (2.1-2.8) |  |

Estimates derived from linear spline models.
