# Supplementary material for: Muscle mass, BMI, and mortality among adults in the United States: A population-based cohort study
Source: PLoS One. 2018 Apr 11;13(4):e0194697. doi: 10.1371/journal.pone.0194697 (PMC5894968; doi:10.1371/journal.pone.0194697)
Supplement: S2 Table — (DOCX) [file pone.0194697.s003.docx]

| **S2 Table. Associations with all-cause mortality in the full cohort and in sensitivity analyses** | | | | | | | |
| --- | --- | --- | --- | --- | --- | --- | --- |
|  | Hazard Ratio (95% CI) | | | | | | |
|  | Body Mass Index (kg/m^2^) | | | | |  |  |
|  | 18.5-<22 | 22-<25 | 25-<30 | 30-<35 | 35-40 | ASMI (per 1 kg/m^2^) | Waist Circumference (per 10 cm) |
|  |  |  |  |  |  |  |  |
| Full cohort (n=11,687) | 1.47 (1.24-1.74) | 1.05 (0.90-1.23) | REF | 1.18 (0.99-1.42) | 1.16 (0.88-1.52) |  |  |
| *With ASMI* | 1.15 (0.94-1.42) | 0.92 (0.78-1.08) | REF | 1.40 (1.11-1.75) | 1.62 (1.14-2.31) | 0.82 (0.73-0.92) |  |
| Subgroup with Waist Circumference Data (n=11,392) | 1.43 (1.20-1.70) | 1.04 (0.89-1.23) | REF | 1.18 (0.98-1.43) | 1.11 (0.83-1.49) |  |  |
| *With ASMI* | 1.11 (0.90-1.38) | 0.91 (0.77-1.07) | REF | 1.40 (1.11-1.78) | 1.57 (1.08-2.29) | 0.81 (0.72-0.92) |  |
| *With Waist Circumference* | 2.02 (1.57-2.59) | 1.26 (1.01-1.58) | REF | 0.98 (0.77-1.24) | 0.75 (0.49-1.16) |  | 1.21 (1.08-1.36) |
| *With ASMI & Waist Circumference* | 1.57 (1.20-2.05) | 1.09 (0.88-1.36) | REF | 1.16 (0.87-1.53) | 1.07 (0.65-1.74) | 0.81 (0.72-0.92) | 1.21 (1.08-1.36) |
| Excluding unintentional weight loss (n=10,867) | 1.43 (1.18-1.72) | 1.02 (0.87-1.20) | REF | 1.18 (0.98-1.43) | 1.14 (0.85-1.54) |  |  |
| *With ASMI* | 1.12 (0.88-1.43) | 0.89 (0.75-1.06) | REF | 1.40 (1.09-1.78) | 1.61 (1.06-2.43) | 0.82 (0.71-0.94) |  |
| Excluding diabetes, CHF, cancer, CKD stage 4 (n=8,802) | 1.49 (1.17-1.91) | 1.07 (0.89-1.28) | REF | 1.33 (1.06-1.67) | 1.13 (0.77-1.67) |  |  |
| *With ASMI* | 1.09 (0.79-1.52) | 0.89 (0.73-1.09) | REF | 1.65 (1.26-2.15) | 1.77 (1.09-2.86) | 0.76 (0.66-0.88) |  |
| Excluding deaths within the first 2 years (n=11,395) | 1.30 (1.07-1.59) | 0.98 (0.83-1.16) | REF | 1.10 (0.91-1.33) | 1.10 (0.82-1.48) |  |  |
| *With ASMI* | 1.06 (0.81-1.37) | 0.87 (0.72-1.05) | REF | 1.27 (0.99-1.63) | 1.48 (0.98-2.22) | 0.84 (0.73-0.97) |  |
| Age <60 (n=7,395) | 1.63 (1.05-2.54) | 1.07 (0.70-1.63) | REF | 1.51 (1.05-2.16) | 1.71 (0.95-3.09) |  |  |
| *With ASMI* | 0.88 (0.53-1.45) | 0.75 (0.51-1.09) | REF | 2.25 (1.38-3.68) | 3.74 (1.67-8.40) | 0.63 (0.48-0.83) |  |
| Age ≥60 (n=4,292) | 1.41 (1.17-1.70) | 1.01 (0.85-1.20) | REF | 1.10 (0.92-1.32) | 1.05 (0.83-1.33) |  |  |
| *With ASMI* | 1.24 (1.01-1.52) | 0.94 (0.77-1.14) | REF | 1.21 (0.98-1.51) | 1.28 (0.95-1.74) | 0.89 (0.80-0.99) |  |
| Age <60, excluding chronic disease (n=6,322) | 1.60 (0.93-2.76) | 1.07 (0.67-1.71) | REF | 1.74 (1.15-2.63) | 1.18 (0.55-2.54) |  |  |
| *With ASMI* | 0.79 (0.42-1.49) | 0.72 (0.45-1.15) | REF | 2.74 (1.60-4.71) | 2.86 (1.06-7.71) | 0.59 (0.45-0.77) |  |
| Age ≥60, excluding chronic disease (n=2,480) | 1.39 (1.07-1.81) | 0.99 (0.80-1.22) | REF | 1.19 (0.91-1.55) | 1.27 (0.89-1.80) |  |  |
| *With ASMI* | 1.19 (0.82-1.72) | 0.90 (0.67-1.20) | REF | 1.34 (1.00-1.80) | 1.63 (1.06-2.50) | 0.86 (0.71-1.05) |  |
| Excluding muscle strengthening activities (n=8,844) | 1.50 (1.27-1.78) | 1.13 (0.94-1.35) | REF | 1.20 (0.99-1.45) | 1.21 (0.90-1.61) |  |  |
| *With ASMI* | 1.16 (0.94-1.44) | 0.97 (0.81-1.17) | REF | 1.44 (1.14-1.82) | 1.75 (1.22-2.51) | 0.80 (0.70-0.91) |  |
| Subgroup not meeting weekly physical activity guidelines (n=5,146) | 1.61 (1.26-2.05) | 1.12 (0.94-1.34) | REF | 1.23 (1.02-1.48) | 1.21 (0.94-1.56) |  |  |
| *With ASMI* | 1.24 (0.94-1.63) | 0.97 (0.80-1.16) | REF | 1.49 (1.18-1.88) | 1.79 (1.24-2.59) | 0.79 (0.68-0.93) |  |
| Adjustment for poverty-income ratio (n=10,679) | 1.44 (1.20-1.72) | 1.02 (0.86-1.21) | REF | 1.20 (1.00-1.44) | 1.21 (0.92-1.60) |  |  |
| *With ASMI* | 1.12 (0.87-1.43) | 0.88 (0.74-1.06) | REF | 1.44 (1.16-1.79) | 1.76 (1.25-2.48) | 0.80 (0.71-0.91) |  |

Abbreviations: ASMI, appendicular skeletal muscle mass index; CHF, congestive heart failure; CKD, chronic kidney disease

Sensitivity analyses to determine the effects of chronic illness, subclinical or undiagnosed disease, and physical activity on our estimates. Unintentional weight loss: excluded participants with unintentional weight loss ≥10 pounds in the prior 12 months; Chronic disease: excluded participants with diabetes, congestive heart failure, or a history of cancer (other than non-melanoma skin cancer) by self-report, or an eGFR<30 mL/min/1.73m^2^; Early mortality: excluding participants who died within the first 2 years of follow-up; Excluding participants who reported not performing muscle strengthening activities; Excluding participants who did not meet minimum weekly physical activity recommendations (≥500 MET-min/wk) based on self-report; Adjusting for the poverty-income ratio as an additional measure of socioeconomic status in the subgroup who reported this information.
