# Supplementary material for: Muscle mass, BMI, and mortality among adults in the United States: A population-based cohort study
Source: PLoS One. 2018 Apr 11;13(4):e0194697. doi: 10.1371/journal.pone.0194697 (PMC5894968; doi:10.1371/journal.pone.0194697)
Supplement: S1 Fig — (PPTX) [file pone.0194697.s004.pptx]

## Slide 1
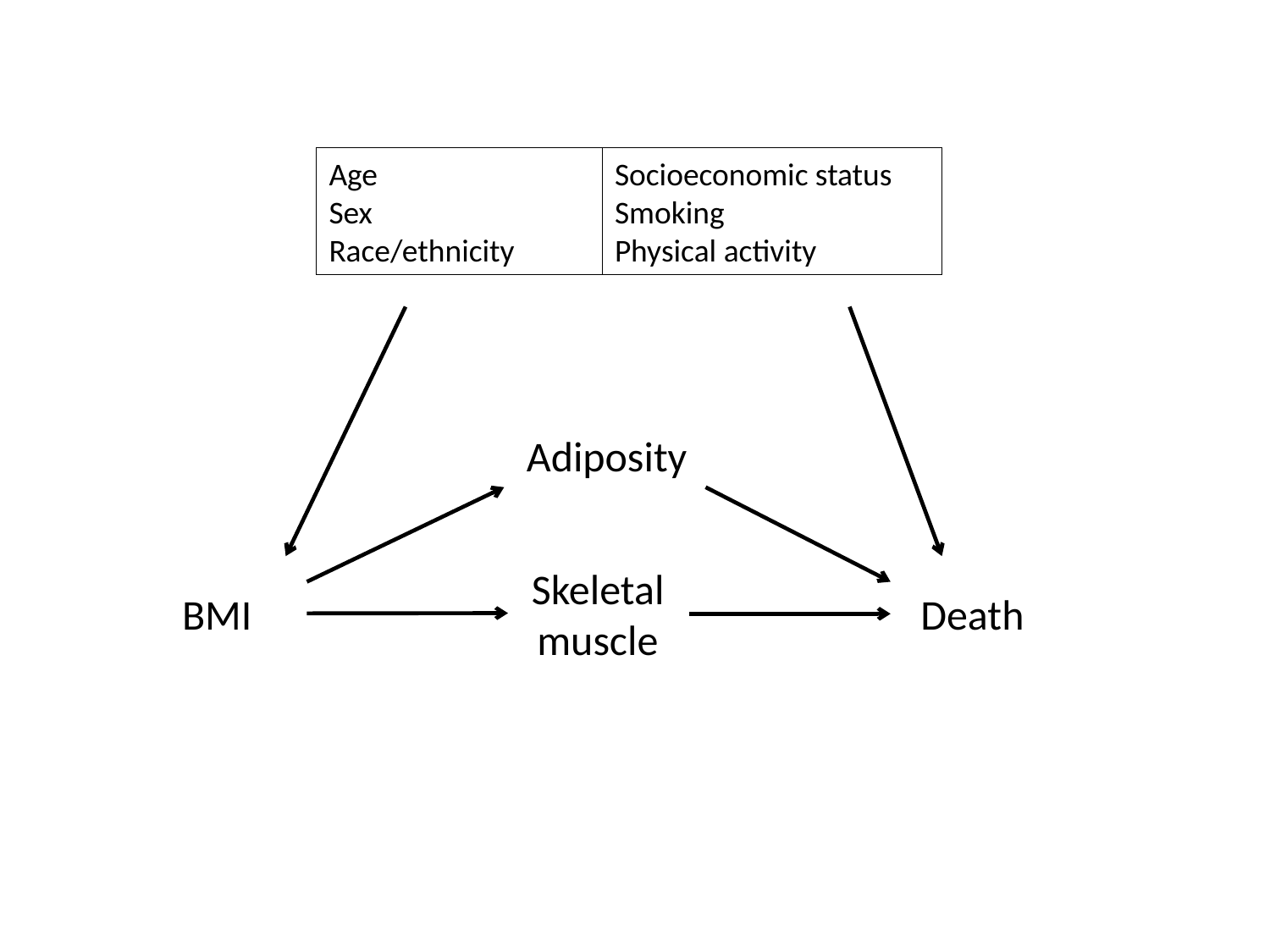

Age
Sex
Race/ethnicity
Socioeconomic status
Smoking
Physical activity
Adiposity
Skeletal muscle
BMI
Death
