# Supplementary figures and images for: Muscle mass, BMI, and mortality among adults in the United States: A population-based cohort study
Source: PLoS One. 2018 Apr 11;13(4):e0194697. doi: 10.1371/journal.pone.0194697 (PMC5894968; doi:10.1371/journal.pone.0194697)

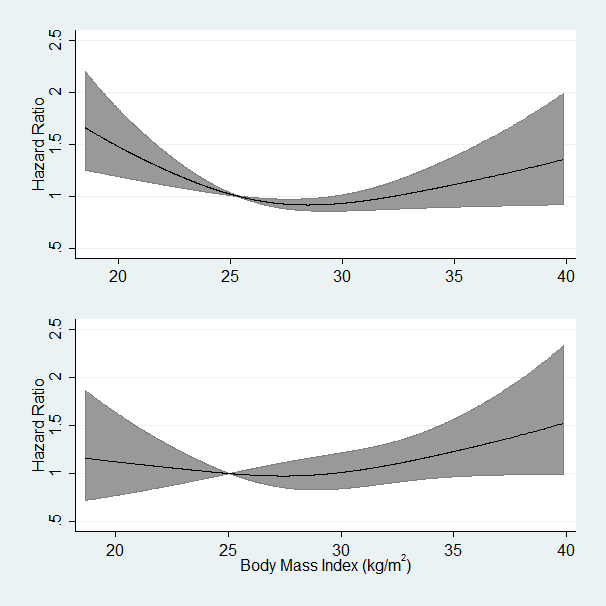

Supplement: S2 Fig — The shaded area represents the 95% confidence interval. (DOCX) [file pone.0194697.s005.docx]

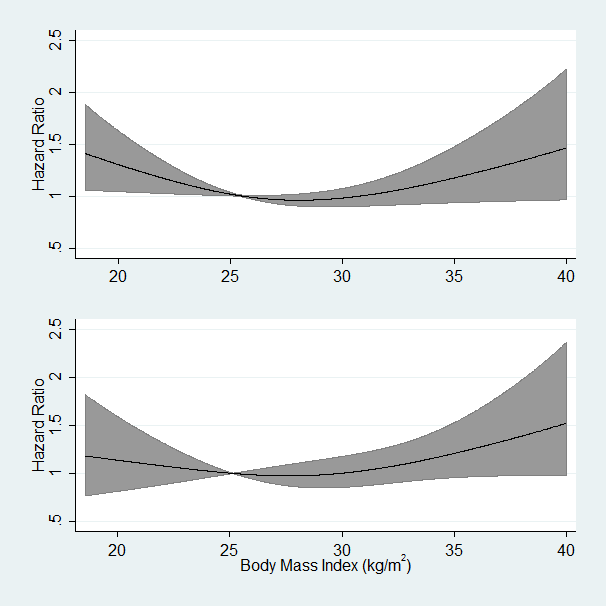

Supplement: S3 Fig — The shaded area represents the 95% confidence interval. (DOCX) [file pone.0194697.s006.docx]

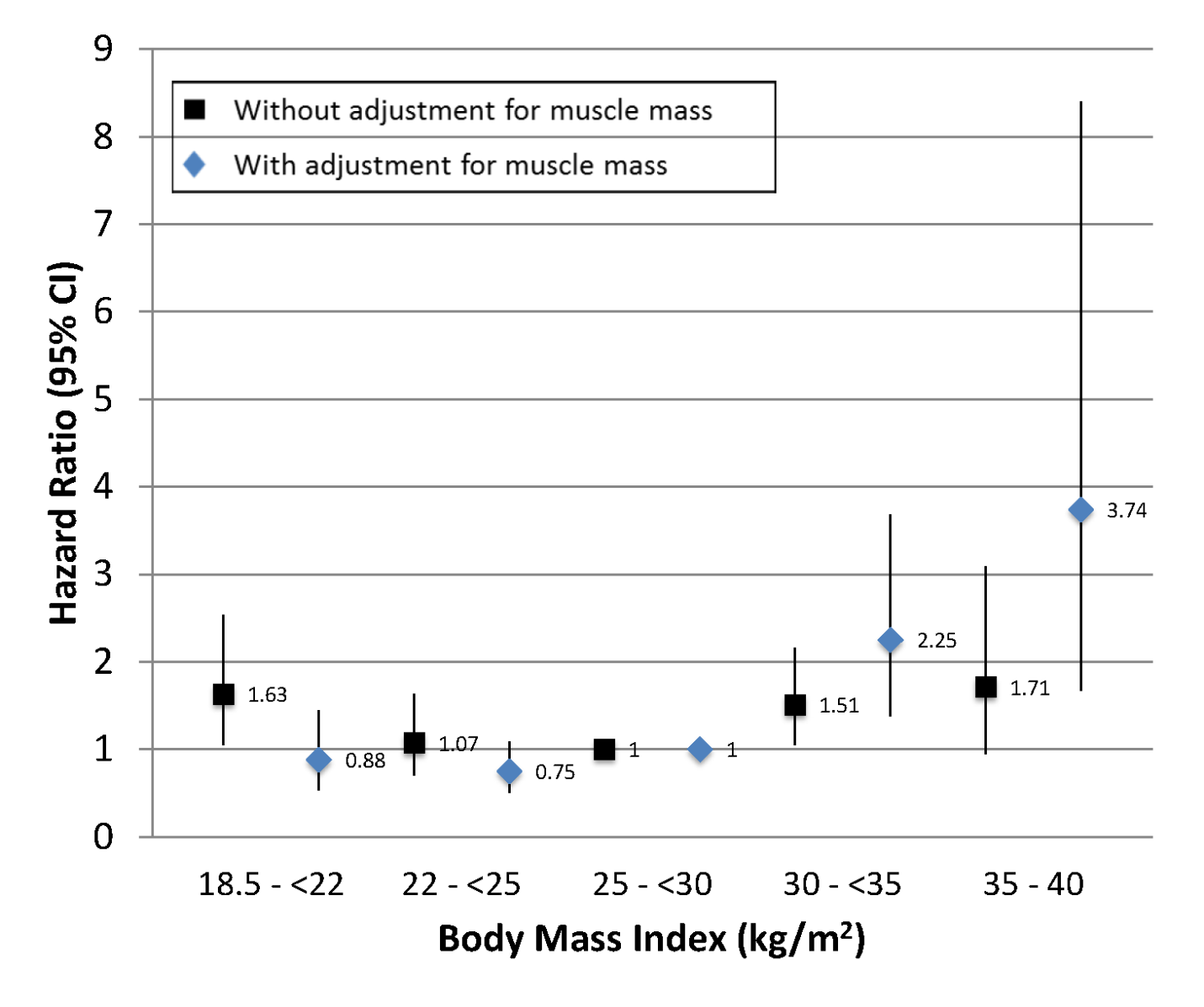

Supplement: S4 Fig — Error bars represent 95% confidence intervals. (DOCX) [file pone.0194697.s007.docx]

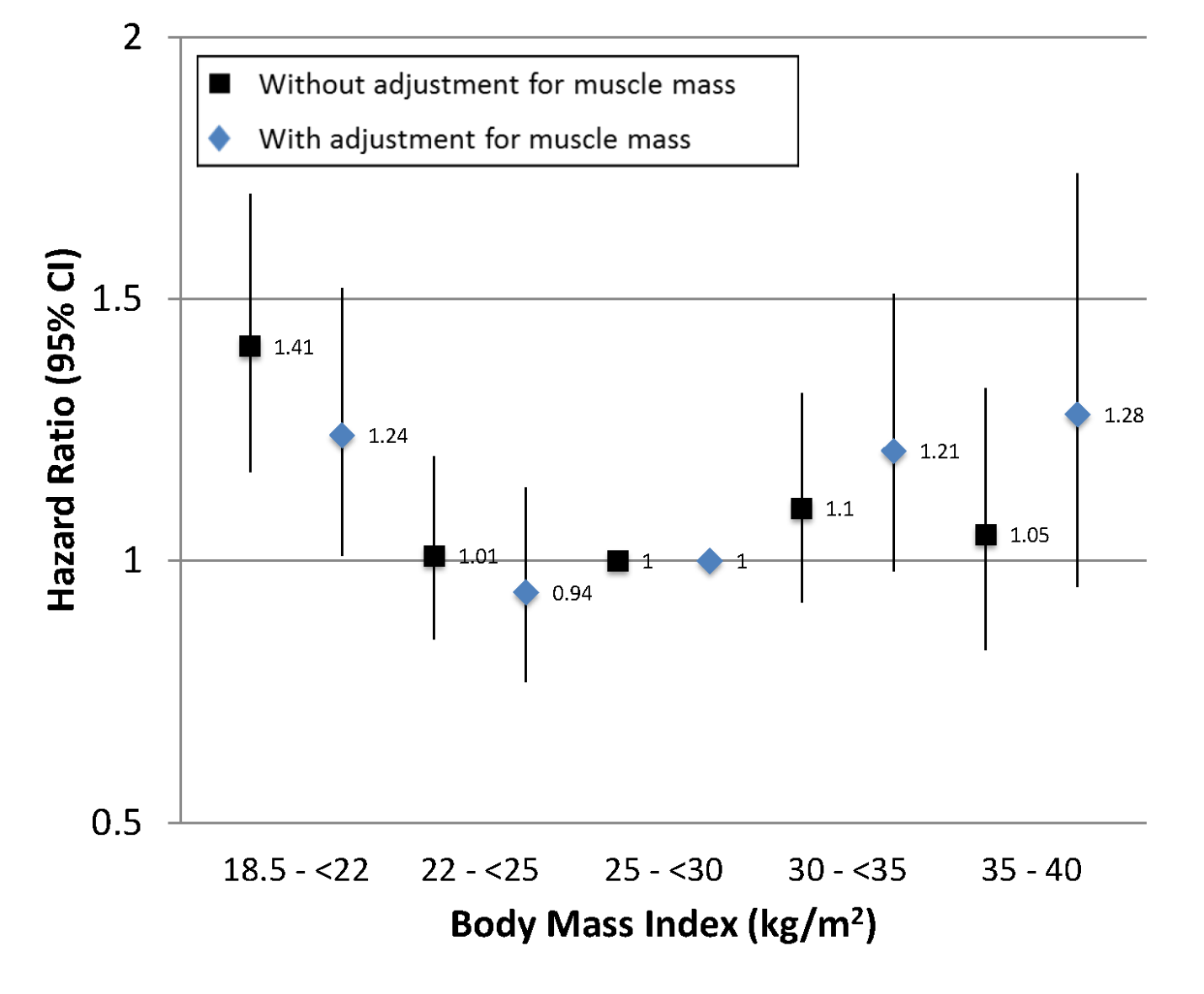

Supplement: S5 Fig — Error bars represent 95% confidence intervals. (DOCX) [file pone.0194697.s008.docx]

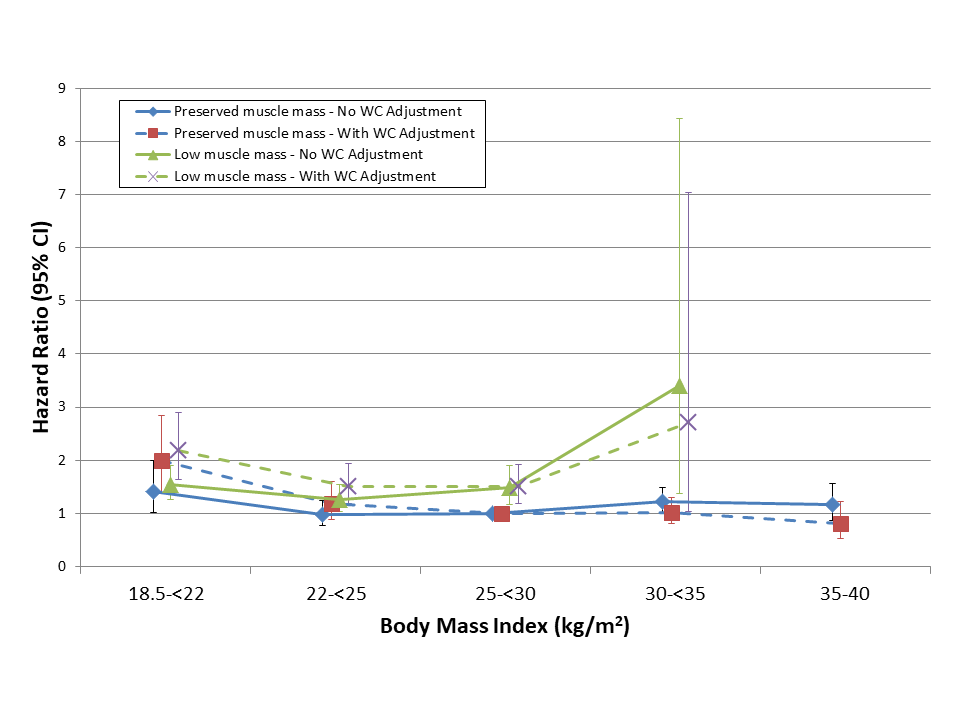

Supplement: S6 Fig — Error bars represent 95% confidence intervals. (DOCX) [file pone.0194697.s009.docx]

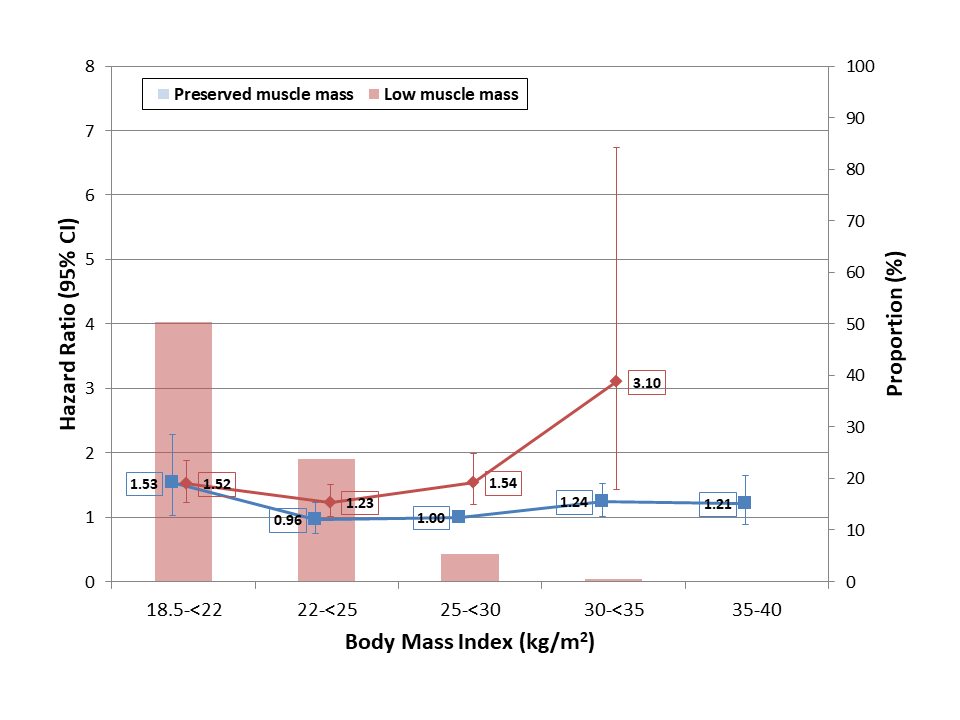

Supplement: S7 Fig — Bars indicate prevalence of low muscle mass in each BMI category. (DOCX) [file pone.0194697.s010.docx]

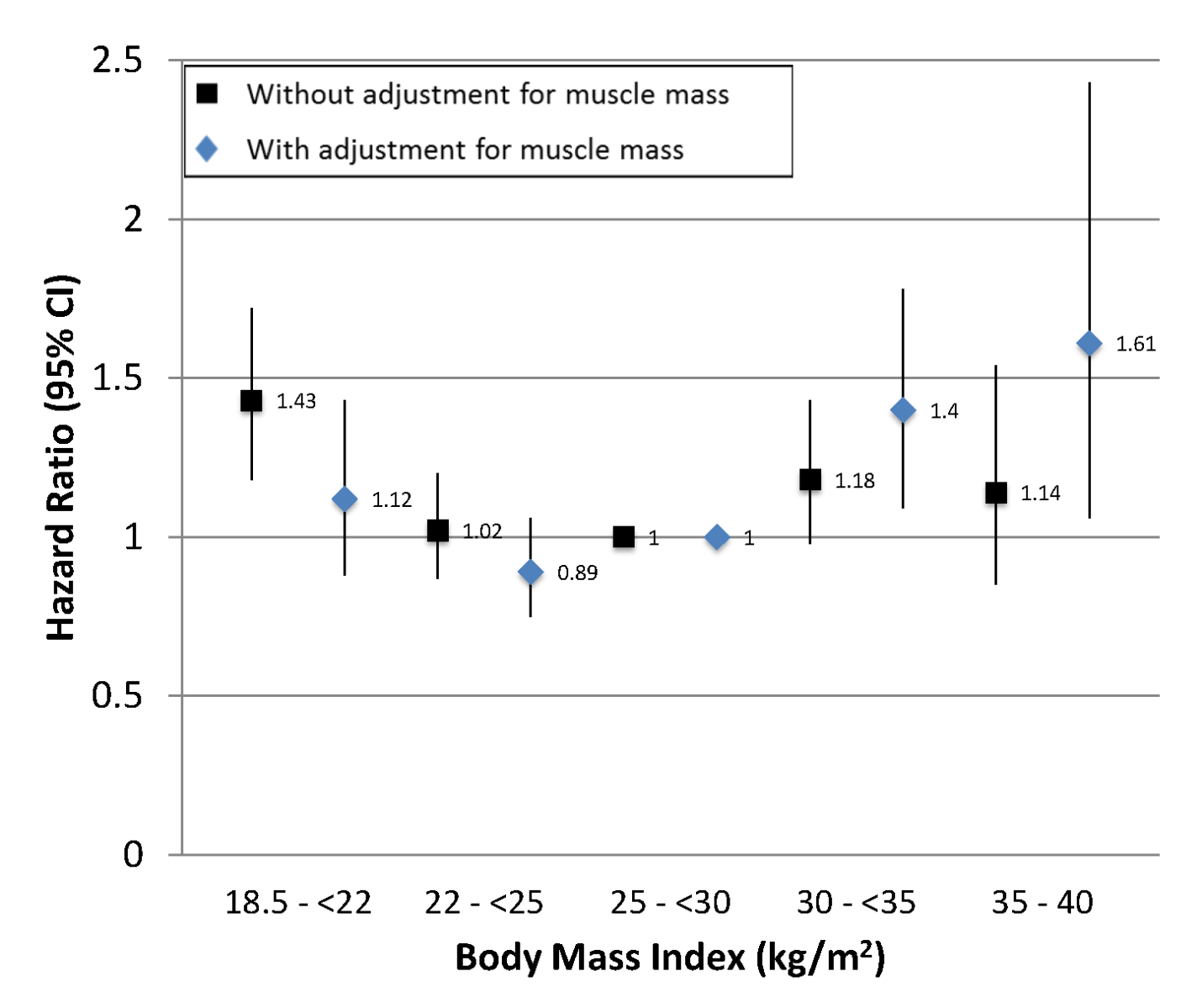

Supplement: S8 Fig — Error bars represent 95% confidence intervals. (DOCX) [file pone.0194697.s011.docx]

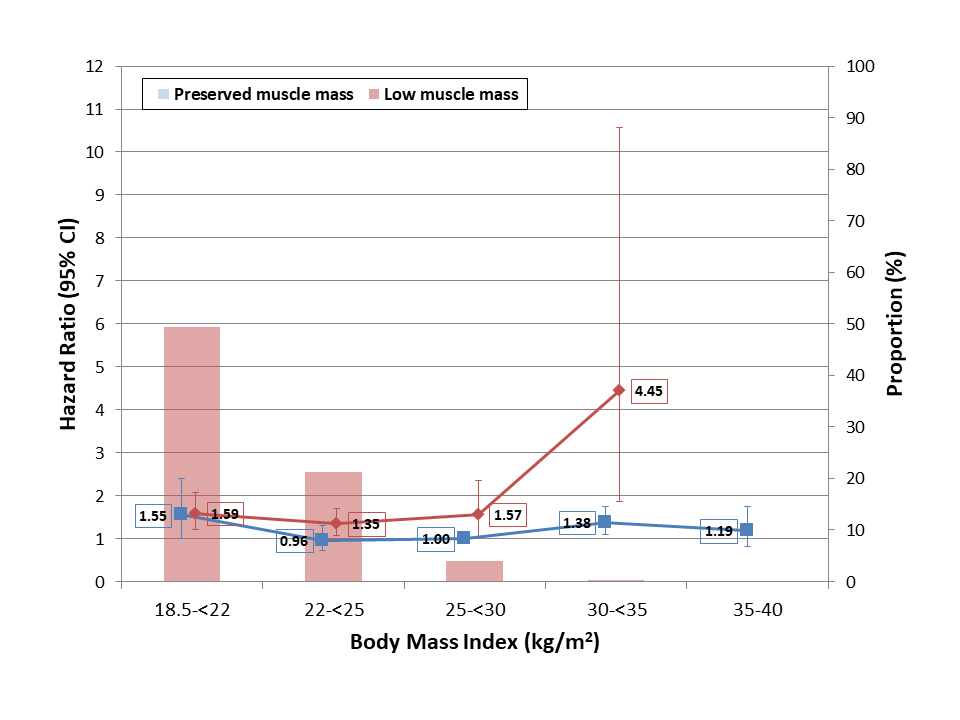

Supplement: S9 Fig — Bars indicate prevalence of low muscle mass in each BMI category. (DOCX) [file pone.0194697.s012.docx]

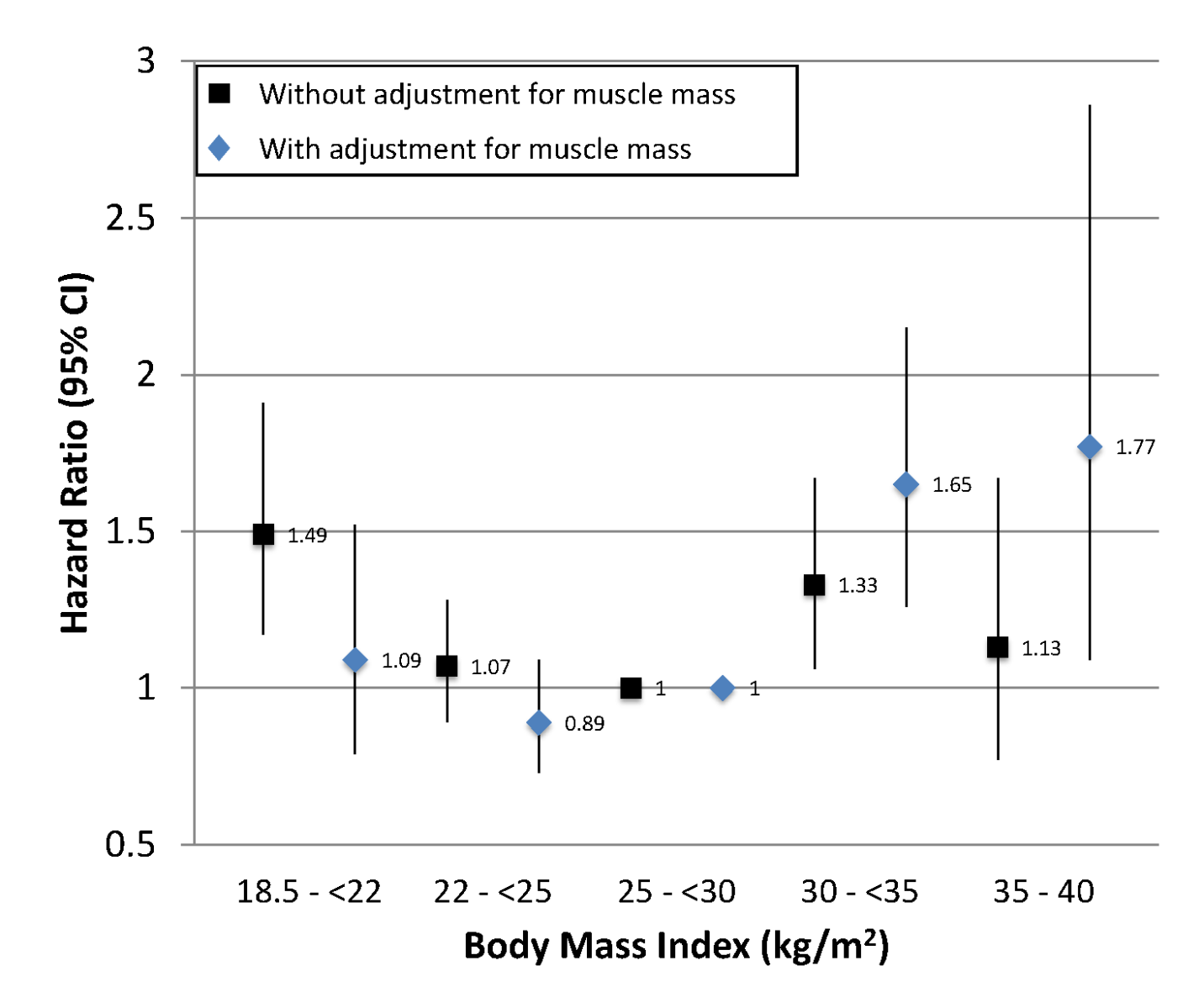

Supplement: S10 Fig — Error bars represent 95% confidence intervals. (DOCX) [file pone.0194697.s013.docx]

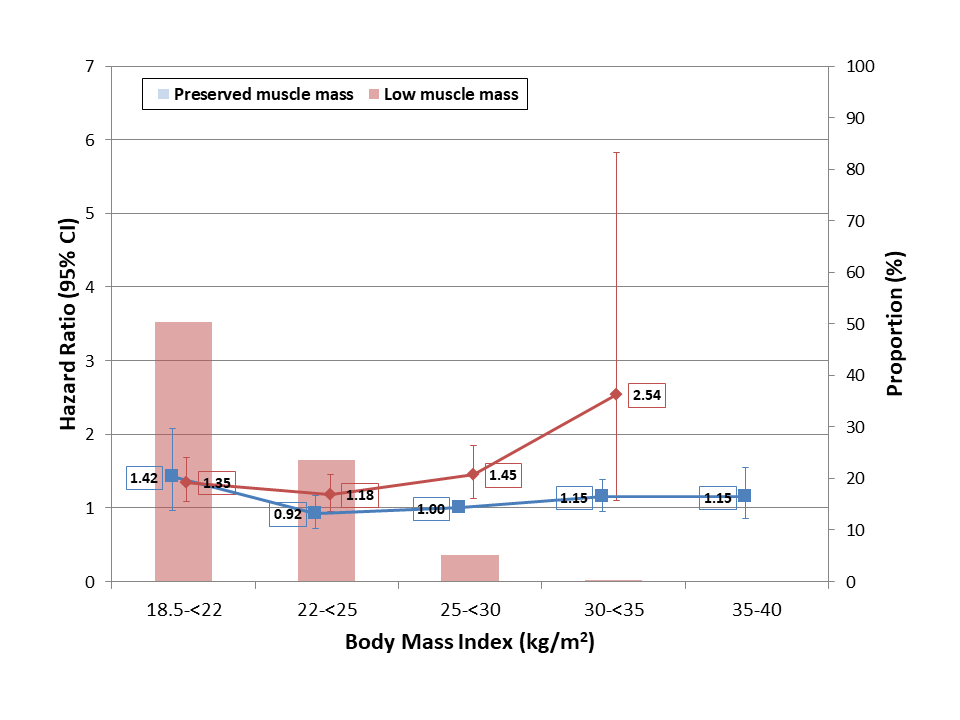

Supplement: S11 Fig — Bars indicate prevalence of low muscle mass in each BMI category. (DOCX) [file pone.0194697.s014.docx]

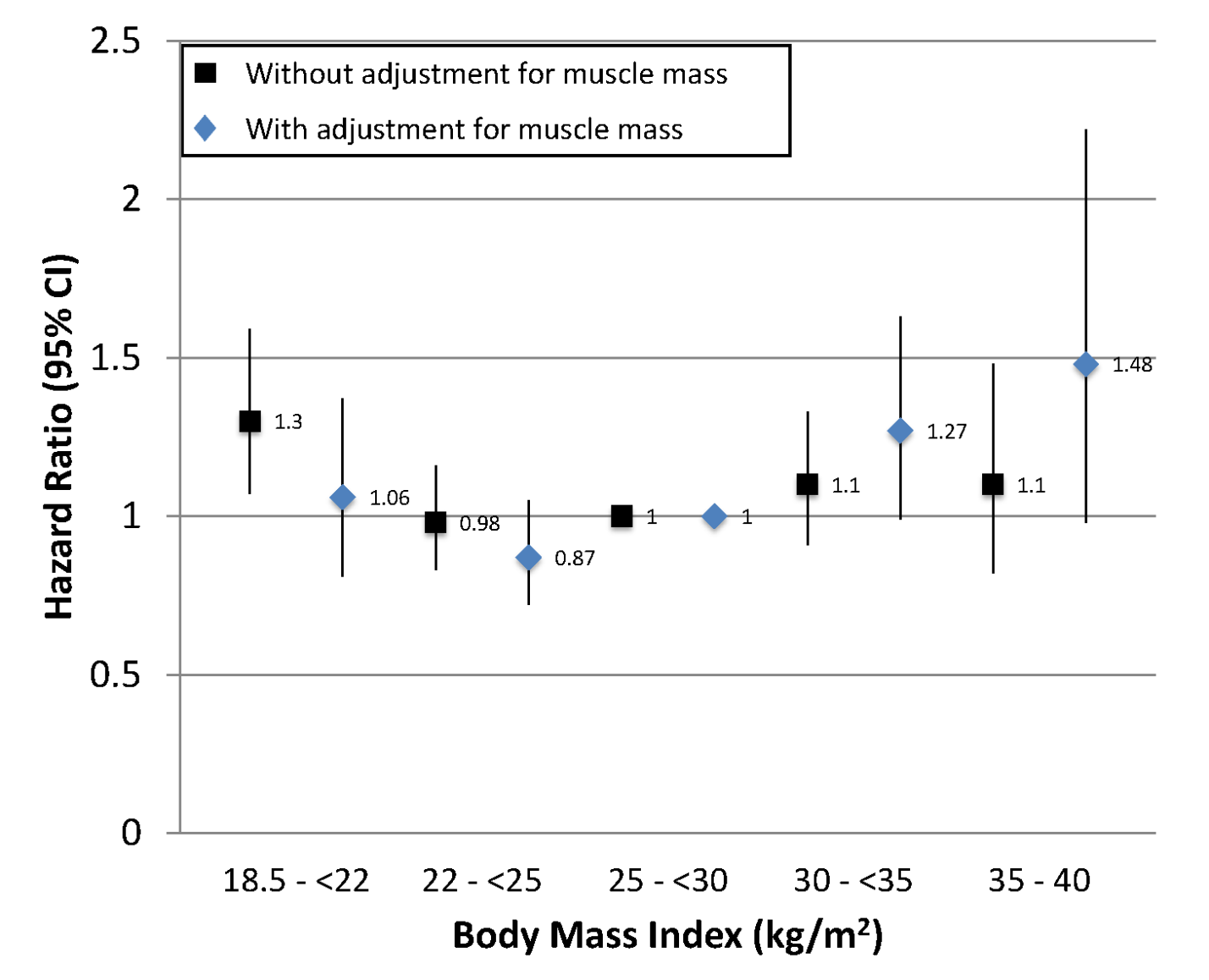

Supplement: S12 Fig — Error bars represent 95% confidence intervals. (DOCX) [file pone.0194697.s015.docx]
